# Normobaric hyperoxia does not improve derangements in diffusion tensor imaging found distant from visible contusions following acute traumatic brain injury

**DOI:** 10.1038/s41598-017-12590-2

**Published:** 2017-09-29

**Authors:** Tonny V. Veenith, Eleanor L. Carter, Julia Grossac, Virginia F. J. Newcombe, Joanne G. Outtrim, Sri Nallapareddy, Victoria Lupson, Marta M. Correia, Marius M. Mada, Guy B. Williams, David K. Menon, Jonathan P. Coles

**Affiliations:** 1 Division of Anaesthesia, University of Cambridge, Addenbrooke’s Hospital, Hills Road, Cambridge, Cambridgeshire CB2 0QQ UK; 2 Department of Critical Care Medicine, University Hospital of Birmingham NHS Trust, Queen Elizabeth Medical Centre, Birmingham, B15 2TH UK; 3 Anesthesiology and Critical Care Department, University Hospital of Toulouse, 31000 Toulouse, France; 4 Wolfson Brain Imaging Centre, Department of Clinical Neurosciences, University of Cambridge, Addenbrooke’s Hospital, Hills Road, Cambridge, CB2 0QQ UK

## Abstract

We have previously shown that normobaric hyperoxia may benefit peri-lesional brain and white matter following traumatic brain injury (TBI). This study examined the impact of brief exposure to hyperoxia using diffusion tensor imaging (DTI) to identify axonal injury distant from contusions. Fourteen patients with acute moderate/severe TBI underwent baseline DTI and following one hour of 80% oxygen. Thirty-two controls underwent DTI, with 6 undergoing imaging following graded exposure to oxygen. Visible lesions were excluded and data compared with controls. We used the 99% prediction interval (PI) for zero change from historical control reproducibility measurements to demonstrate significant change following hyperoxia. Following hyperoxia DTI was unchanged in controls. In patients following hyperoxia, mean diffusivity (MD) was unchanged despite baseline values lower than controls (p < 0.05), and fractional anisotropy (FA) was lower within the left uncinate fasciculus, right caudate and occipital regions (p < 0.05). 16% of white and 14% of mixed cortical and grey matter patient regions showed FA decreases greater than the 99% PI for zero change. The mechanistic basis for some findings are unclear, but suggest that a short period of normobaric hyperoxia is not beneficial in this context. Confirmation following a longer period of hyperoxia is required.

## Introduction

While normobaric hyperoxia (NH) has been used to increase brain tissue oxygen partial pressure (BptO_2_) following traumatic brain injury (TBI) it is not routine therapy. Reductions in BtpO_2_ are associated with worse outcome^1,2^, and interventions aimed at optimising oxygen delivery have shown benefit^1,3,4^. ^15^O positron emission tomography (^15^O PET) has been used to show that NH can improve oxygen utilisation in “at-risk” regions of metabolically compromised tissue in pericontusional and white matter regions^4^. In addition, evidence obtained using diffusion tensor imaging (DTI) show how cytotoxic oedema within a rim of pericontusional tissue can be ameliorated with a short NH intervention^5^.

These results are in conflict with evidence demonstrating increases in microdialysis glutamate^6^, and studies showing an association between arterial hyperoxia and poor outcome following severe TBI^7^. These highlight the potential deleterious effects on pulmonary function and worsening neuronal injury due to oxidative stress^8,9^. Given this background, it is clear that further study of the regional effects of normobaric hyperoxia across the injured brain is warranted to ensure it is used appropriately.

Diffusion tensor imaging has been used to demonstrate evidence of traumatic axonal injury following TBI even when conventional imaging appears normal^10^. Imaging findings are dynamic and potentially reversible^11,12^, suggesting that DTI could be used as a biomarker of the effectiveness of therapeutic interventions in TBI. In this study we aimed to address the impact of NH distant from visible contusions using novel data from a cohort of subjects previously included within a publication by Veenith *et al*.^5^. Such data should help inform the design and conduct of any future clinical trial of this intervention in TBI.

## Results

### Effect of graduated oxygen therapy on diffusion tensor imaging in healthy volunteers

The DTI data at each level of inspired oxygen are displayed in Tables 1 and 2 for white and mixed cortical and deep grey matter regions respectively. As expected, there were significant differences between the brain regions for fractional anisotropy (FA), axial diffusivity (AD), radial diffusivity (RD) and mean diffusivity (MD) (p < 0.001 for all comparisons using ANOVA with Bonferoni correction). While the DTI parameters were variable across the different brain regions there were no significant changes in FA, AD, RD and MD with an increase in the fraction of inspired oxygen (FiO_2_) within white matter (p = 0.82, 0.87, 0.70 and 0.68 respectively, analysis of variance (ANOVA)) and mixed cortical and deep grey matter regions of interest (ROIs) (0.66, 0.32, 0.47 and 0.40 respectively, ANOVA).

**Table 1:** Impact of oxygen therapy on diffusion tensor imaging parameters in healthy volunteers within white matter regions.

	*FA*			*AD*			*RD*			*MD*		
	21%	60%	100%	21%	60%	100%	21%	60%	100%	21%	60%	100%
ACC	0.50221 ± 0.14227	0.51701 ± 0.13994	0.51598 ± 0.12946	0.00147 ± 0.00014	0.00151 ± 0.00012	0.00153 ± 0.00010	0.00063 ± 0.00014	0.00063 ± 0.00017	0.00065 ± 0.00014	0.00091 ± 0.00007	0.00093 ± 0.00009	0.00094 ± 0.00006
BCC	0.62040 ± 0.18813	0.61152 ± 0.17453	0.60411 ± 0.18935	0.00163 ± 0.00021	0.00163 ± 0.00019	0.00162 ± 0.00020	0.00054 ± 0.00019	0.00056 ± 0.00018	0.00057 ± 0.00021	0.00091 ± 0.00010	0.00091 ± 0.00008	0.00092 ± 0.00012
PCC	0.72065 ± 0.06245	0.70915 ± 0.06939	0.72249 ± 0.05558	0.00169 ± 0.00018	0.00169 ± 0.00018	0.00166 ± 0.00013	0.00045 ± 0.00019	0.00046 ± 0.00018	0.00041 ± 0.00010	0.00086 ± 0.00018	0.00087 ± 0.00018	0.00083 ± 0.00010
ATR left	0.41352 ± 0.01627	0.41092 ± 0.01820	0.41295 ± 0.01898	0.00116 ± 0.00002	0.00116 ± 0.00002	0.00117 ± 0.00002	0.00061 ± 0.00002	0.00061 ± 0.00002	0.00062 ± 0.00003	0.00079 ± 0.00002	0.00080 ± 0.00002	0.00080 ± 0.00002
ATR right	0.38022 ± 0.01080	0.38072 ± 0.01289	0.37752 ± 0.01495	0.00123 ± 0.00002	0.00123 ± 0.00002	0.00124 ± 0.00002	0.00071 ± 0.00002	0.00071 ± 0.00003	0.00072 ± 0.00003	0.00088 ± 0.00002	0.00089 ± 0.00003	0.00090 ± 0.00003
SLF left	0.35047 ± 0.00847	0.34760 ± 0.01557	0.34740 ± 0.01195	0.00113 ± 0.00003	0.00113 ± 0.00003	0.00113 ± 0.00003	0.00069 ± 0.00003	0.00069 ± 0.00003	0.00070 ± 0.00003	0.00083 ± 0.00003	0.00084 ± 0.00003	0.00084 ± 0.00003
SLF right	0.37194 ± 0.01291	0.37352 ± 0.01132	0.37423 ± 0.01091	0.00114 ± 0.00003	0.00115 ± 0.00002	0.00114 ± 0.00002	0.00066 ± 0.00002	0.00067 ± 0.00002	0.00067 ± 0.00002	0.00082 ± 0.00002	0.00083 ± 0.00002	0.00083 ± 0.00002
ILF left	0.39235 ± 0.00718	0.39099 ± 0.01296	0.39078 ± 0.01037	0.00116 ± 0.00003	0.00116 ± 0.00004	0.00116 ± 0.00005	0.00062 ± 0.00002	0.00063 ± 0.00003	0.00062 ± 0.00003	0.00080 ± 0.00002	0.00080 ± 0.00003	0.00080 ± 0.00004
ILF right	0.41145 ± 0.02079	0.40903 ± 0.02208	0.41097 ± 0.01977	0.00122 ± 0.00003	0.00122 ± 0.00003	0.00123 ± 0.00003	0.00064 ± 0.00003	0.00064 ± 0.00003	0.00064 ± 0.00003	0.00083 ± 0.00003	0.00083 ± 0.00003	0.00084 ± 0.00002
C left	0.31285 ± 0.04658	0.31081 ± 0.04683	0.31204 ± 0.05076	0.00117 ± 0.00005	0.00117 ± 0.00004	0.00116 ± 0.00005	0.00072 ± 0.00005	0.00073 ± 0.00005	0.00072 ± 0.00005	0.00087 ± 0.00003	0.00088 ± 0.00002	0.00087 ± 0.00002
C right	0.31179 ± 0.07048	0.30697 ± 0.07175	0.31259 ± 0.06668	0.00131 ± 0.00007	0.00132 ± 0.00008	0.00131 ± 0.00006	0.00084 ± 0.00012	0.00085 ± 0.00012	0.00083 ± 0.00013	0.00100 ± 0.00009	0.00100 ± 0.00009	0.00099 ± 0.00010
UF left	0.39901 ± 0.01733	0.39133 ± 0.02412	0.39329 ± 0.01869	0.00115 ± 0.00003	0.00115 ± 0.00004	0.00115 ± 0.00004	0.00061 ± 0.00003	0.00062 ± 0.00004	0.00062 ± 0.00004	0.00079 ± 0.00003	0.00080 ± 0.00003	0.00080 ± 0.00004
UF right	0.39146 ± 0.03456	0.39149 ± 0.03235	0.38938 ± 0.03364	0.00124 ± 0.00005	0.00124 ± 0.00005	0.00125 ± 0.00004	0.00068 ± 0.00005	0.00068 ± 0.00006	0.00069 ± 0.00005	0.00087 ± 0.00004	0.00087 ± 0.00005	0.00088 ± 0.00004
CT left	0.48057 ± 0.01368	0.47754 ± 0.00970	0.47619 ± 0.01439	0.00130 ± 0.00003	0.00129 ± 0.00003	0.00130 ± 0.00003	0.00064 ± 0.00004	0.00064 ± 0.00004	0.00065 ± 0.00004	0.00086 ± 0.00004	0.00086 ± 0.00003	0.00087 ± 0.00004
CT right	0.48926 ± 0.00934	0.48504 ± 0.00208	0.48648 ± 0.00921	0.00126 ± 0.00004	0.00126 ± 0.00004	0.00127 ± 0.00003	0.00059 ± 0.00004	0.00060 ± 0.00004	0.00060 ± 0.00003	0.00081 ± 0.00004	0.00082 ± 0.00004	0.00082 ± 0.00003
F Mi	0.36611 ± 0.01964	0.36525 ± 0.02001	0.36728 ± 0.01612	0.00122 ± 0.00003	0.00122 ± 0.00004	0.00124 ± 0.00002	0.00071 ± 0.00004	0.00071 ± 0.00004	0.00072 ± 0.00003	0.00088 ± 0.00003	0.00088 ± 0.00004	0.00089 ± 0.00003
F Ma	0.41639 ± 0.03417	0.41371 ± 0.04138	0.41553 ± 0.03673	0.00130 ± 0.00006	0.00130 ± 0.00006	0.00130 ± 0.00005	0.00068 ± 0.00005	0.00068 ± 0.00005	0.00068 ± 0.00005	0.00088 ± 0.00004	0.00089 ± 0.00004	0.00088 ± 0.00003
VM	0.55563 ± 0.05028	0.54126 ± 0.04962	0.53096 ± 0.05866	0.00139 ± 0.00017	0.00137 ± 0.00021	0.00141 ± 0.00021	0.00049 ± 0.00019	0.00050 ± 0.00019	0.00054 ± 0.00025	0.00079 ± 0.00018	0.00079 ± 0.00020	0.00083 ± 0.00024
DM	0.51031 ± 0.05171	0.50918 ± 0.05721	0.49917 ± 0.05955	0.00124 ± 0.00006	0.00125 ± 0.00005	0.00128 ± 0.00008	0.00054 ± 0.00009	0.00054 ± 0.00008	0.00058 ± 0.00009	0.00077 ± 0.00007	0.00078 ± 0.00007	0.00081 ± 0.00008
CP left	0.50069 ± 0.02066	0.50010 ± 0.01832	0.48834 ± 0.01827	0.00112 ± 0.00009	0.00112 ± 0.00008	0.00112 ± 0.00006	0.00047 ± 0.00005	0.00048 ± 0.00005	0.00049 ± 0.00003	0.00069 ± 0.00006	0.00069 ± 0.00006	0.00070 ± 0.00004
CP right	0.52664 ± 0.01868	0.52488 ± 0.01068	0.52953 ± 0.02562	0.00115 ± 0.00006	0.00115 ± 0.00007	0.00113 ± 0.00008	0.00046 ± 0.00001	0.00046 ± 0.00002	0.00045 ± 0.00003	0.00069 ± 0.00003	0.00069 ± 0.00004	0.00068 ± 0.00004
P left	0.54257 ± 0.03019	0.52804 ± 0.04279	0.53039 ± 0.03928	0.00128 ± 0.00007	0.00127 ± 0.00010	0.00127 ± 0.00014	0.00051 ± 0.00006	0.00052 ± 0.00007	0.00052 ± 0.00008	0.00077 ± 0.00006	0.00077 ± 0.00008	0.00077 ± 0.00010
P right	0.47292 ± 0.02088	0.45338 ± 0.04297	0.46720 ± 0.02883	0.00125 ± 0.00010	0.00126 ± 0.00016	0.00126 ± 0.00013	0.00059 ± 0.00007	0.00061 ± 0.00012	0.00060 ± 0.00010	0.00081 ± 0.00008	0.00083 ± 0.00013	0.00082 ± 0.00011
Mean	0.45389 ± 0.11151	0.44998 ± 0.10941	0.45021 ± 0.10944	0.00127 ± 0.00017	0.00127 ± 0.00017	0.00128 ± 0.00017	0.00061 ± 0.00012	0.00062 ± 0.00013	0.00062 ± 0.00013	0.00083 ± 0.00010	0.00084 ± 0.00010	0.00084 ± 0.00010

Data are mean ± standard deviation using the atlas regions of interest applied in normalised space for fractional anisotropy (FA), mean diffusivity (MD) mm^2^/second, axial (AD) mm^2^/second and radial diffusivity (RD) mm^2^/second for six volunteers. Anterior corpus callosum (ACC), body corpus callosum (BCC), posterior corpus callosum (PCC), anterior thalamic radiation (ATR), superior longitudinal fasciculus (SLF), inferior longitudinal fasciculus (ILF), Cingulum (C), uncinate fasciculus (UF), corticospinal tract (CT), forceps minor (F Mi), forceps major (F Ma), ventral midbrain (VM), dorsal midbrain (DM), cerebral peduncle (CP), pons (P).

**Table 2:** Impact of oxygen therapy on diffusion tensor imaging parameters in healthy volunteers within mixed cortical and deep grey matter regions.

	*FA*			*AD*			*RD*			*MD*		
	21%	60%	100%	21%	60%	100%	21%	60%	100%	21%	60%	100%
Caud left	0.31673 ± 0.02508	0.30618 ± 0.01805	0.30588 ± 0.02167	0.00098 ± 0.00004	0.00098 ± 0.00005	0.00098 ± 0.00005	0.00060 ± 0.00002	0.00062 ± 0.00002	0.00062 ± 0.00004	0.00073 ± 0.00002	0.00074 ± 0.00003	0.00074 ± 0.00004
Caud right	0.25826 ± 0.01186	0.26358 ± 0.01362	0.26141 ± 0.01089	0.00100 ± 0.00005	0.00100 ± 0.00005	0.00101 ± 0.00005	0.00070 ± 0.00003	0.00070 ± 0.00004	0.00070 ± 0.00001	0.00080 ± 0.00003	0.00080 ± 0.00004	0.00081 ± 0.00002
Thal left	0.34176 ± 0.01511	0.34810 ± 0.01540	0.35057 ± 0.01520	0.00102 ± 0.00003	0.00103 ± 0.00003	0.00103 ± 0.00002	0.00062 ± 0.00002	0.00061 ± 0.00001	0.00061 ± 0.00001	0.00075 ± 0.00002	0.00075 ± 0.00001	0.00075 ± 0.00001
Thal right	0.33759 ± 0.01271	0.34540 ± 0.00680	0.33842 ± 0.01065	0.00107 ± 0.00004	0.00108 ± 0.00001	0.00108 ± 0.00002	0.00066 ± 0.00002	0.00066 ± 0.00002	0.00067 ± 0.00001	0.00080 ± 0.00002	0.00080 ± 0.00001	0.00081 ± 0.00002
H left	0.27931 ± 0.01450	0.28370 ± 0.01449	0.28669 ± 0.01327	0.00130 ± 0.00005	0.00131 ± 0.00005	0.00132 ± 0.00006	0.00088 ± 0.00005	0.00089 ± 0.00004	0.00089 ± 0.00005	0.00102 ± 0.00004	0.00103 ± 0.00004	0.00103 ± 0.00005
H right	0.28173 ± 0.01553	0.28166 ± 0.01753	0.28907 ± 0.01828	0.00144 ± 0.00005	0.00146 ± 0.00007	0.00146 ± 0.00005	0.00098 ± 0.00005	0.00099 ± 0.00006	0.00099 ± 0.00004	0.00113 ± 0.00005	0.00115 ± 0.00006	0.00115 ± 0.00004
F left	0.24207 ± 0.00387	0.24104 ± 0.00709	0.24324 ± 0.00745	0.00124 ± 0.00008	0.00126 ± 0.00008	0.00125 ± 0.00007	0.00092 ± 0.00007	0.00093 ± 0.00007	0.00092 ± 0.00006	0.00102 ± 0.00008	0.00104 ± 0.00007	0.00103 ± 0.00006
F right	0.23650 ± 0.00423	0.23606 ± 0.00211	0.23572 ± 0.00646	0.00128 ± 0.00007	0.00128 ± 0.00007	0.00128 ± 0.00005	0.00096 ± 0.00006	0.00095 ± 0.00006	0.00096 ± 0.00005	0.00106 ± 0.00006	0.00106 ± 0.00006	0.00106 ± 0.00005
P left	0.25733 ± 0.00825	0.25441 ± 0.01647	0.25662 ± 0.01468	0.00126 ± 0.00011	0.00127 ± 0.00010	0.00127 ± 0.00010	0.00091 ± 0.00010	0.00092 ± 0.00009	0.00092 ± 0.00009	0.00103 ± 0.00010	0.00104 ± 0.00009	0.00104 ± 0.00009
P right	0.25676 ± 0.00769	0.25622 ± 0.01135	0.25826 ± 0.01062	0.00129 ± 0.00007	0.00130 ± 0.00007	0.00130 ± 0.00006	0.00094 ± 0.00006	0.00095 ± 0.00006	0.00095 ± 0.00006	0.00106 ± 0.00006	0.00107 ± 0.00006	0.00106 ± 0.00006
Temp left	0.23648 ± 0.00696	0.23727 ± 0.01211	0.23691 ± 0.00850	0.00109 ± 0.00003	0.00111 ± 0.00004	0.00111 ± 0.00003	0.00077 ± 0.00003	0.00078 ± 0.00004	0.00079 ± 0.00003	0.00088 ± 0.00003	0.00089 ± 0.00004	0.00090 ± 0.00003
Temp right	0.25081 ± 0.00664	0.25316 ± 0.00908	0.25276 ± 0.00909	0.00118 ± 0.00002	0.00119 ± 0.00002	0.00120 ± 0.00002	0.00083 ± 0.00002	0.00084 ± 0.00002	0.00084 ± 0.00003	0.00094 ± 0.00002	0.00096 ± 0.00002	0.00096 ± 0.00003
O left	0.24035 ± 0.01366	0.24140 ± 0.01701	0.24371 ± 0.01533	0.00115 ± 0.00005	0.00116 ± 0.00004	0.00116 ± 0.00004	0.00083 ± 0.00005	0.00084 ± 0.00004	0.00084 ± 0.00005	0.00094 ± 0.00005	0.00095 ± 0.00004	0.00095 ± 0.00005
O right	0.23499 ± 0.01004	0.23343 ± 0.01326	0.23542 ± 0.01063	0.00118 ± 0.00005	0.00119 ± 0.00003	0.00119 ± 0.00003	0.00086 ± 0.00004	0.00087 ± 0.00003	0.00087 ± 0.00003	0.00096 ± 0.00004	0.00098 ± 0.00003	0.00097 ± 0.00003
Cereb left	0.22897 ± 0.01667	0.22951 ± 0.01207	0.23166 ± 0.01633	0.00103 ± 0.00009	0.00104 ± 0.00009	0.00106 ± 0.00008	0.00074 ± 0.00009	0.00075 ± 0.00009	0.00076 ± 0.00008	0.00084 ± 0.00009	0.00085 ± 0.00009	0.00086 ± 0.00008
Cereb right	0.22549 ± 0.01729	0.22826 ± 0.01589	0.22658 ± 0.01788	0.00102 ± 0.00007	0.00103 ± 0.00008	0.00103 ± 0.00008	0.00074 ± 0.00007	0.00075 ± 0.00009	0.00075 ± 0.00008	0.00083 ± 0.00007	0.00085 ± 0.00009	0.00084 ± 0.00008
Mean	0.26407 ± 0.03850	0.26496 ± 0.03954	0.26581 ± 0.03910	0.00116 ± 0.00014	0.00117 ± 0.00014	0.00117 ± 0.00014	0.00081 ± 0.00013	0.00082 ± 0.00013	0.00082 ± 0.00013	0.00092 ± 0.00013	0.00093 ± 0.00013	0.00093 ± 0.00013

Data are mean ± standard deviation using the atlas regions of interest applied in normalised space for fractional anisotropy (FA), mean diffusivity (MD) mm^2^/second, axial (AD) mm^2^/second and radial diffusivity (RD) mm^2^/second for six volunteers. Caudate (Caud), thalamus (Thal), hippocampus (H), frontal (F), parietal (P), temporal (Temp), occipital (O), cerebellum (Cereb).

### Diffusion tensor imaging in patients and healthy volunteers

Patient characteristics are shown in Table 3. For the 14 patients and 32 healthy volunteers there was no significant difference in age (p = 0.48, Mann-Whitney U test). The baseline ROI data for healthy volunteers and normoxic patients from predominantly white matter, and mixed cortical and deep grey matter are summarised in Tables 4 and 5 respectively. These demonstrate that baseline patient data show lower FA, MD, AD and RD values than healthy volunteers in a variety of normal appearing white and mixed cortical and deep grey matter regions (p < 0.05, unpaired t tests with Bonferroni correction).

**Table 3:** Patient characteristics.

Subject	Age	Sex	Mechanism	Injury	Parenchymal lesion volume (ml)	DAI	GCS	Marshall score	APACHE II	ISS	Neurosurgery	Second tier therapies	Days to MRI	GOS
1	53	M	RTA	Bitemporal, basal ganglia& cortical contusions. Bilateral frontal SDH	100	Yes	4	NEML	17	34			4	MD
2	34	M	RTA	Bilateral subcortical & deep white matter, corpus callosum, R thalamus, midbrain & cerebellar contusions. IVH, L occipital & fronto-temporal SDH	20	Yes	4	NEML	21	20	EVD		3	VS
3	34	M	Assault	Bilateral frontal, temporal, R occipital, thalamus & L cerebellar contusions. IVH	607	No	8	EML	25	16	DC, R SDH EVD		3	SD
4	21	M	RTA	Bilateral cortical, corpus callosum, dorsal midbrain & pons contusions	46	Yes	10	NEML	21	50		H	2	MD
5	31	M	RTA	Bilateral frontal, temporal & L occipito-parietal & midbrain contusions	259	No	6	EML	17	29	DC, R SDH		1	MD
6	29	M	Assault	Bilateral frontal & temporal contusions. Bilateral temporal SDH	444	No	10	EML	17	16	DC, EVD	H	2	GR
7	58	M	Fall	Bilateral frontal, temporal & R parietal contusions. Bifrontal SDH & tSAH	122	No	10	NEML	20	34			4	GR
8	26	M	RTA	Bilateral frontal & temporal contusions. R temporal & L frontotemporal SDH	346	No	3	NEML	17	75			3	MD
9	28	M	Assault	R frontotemporal contusions &R SDH	38	No	12	EML	24	36	DC		3	GR
10	61	M	Fall	Bilateral frontal & temporal, corpus callosum & midbrain contusions. L SDH & IVH	358	No	5	NEML	22	75			9	NA
11	60	M	Fall	L Frontal, Temporal & Parietal contusions. L SDH	236	No	14	NEML	8	34			3	MD
12	31	F	Fall	R frontal, temporal, parietal, occipital, bilateral thalamic & midbrain contusions R SDH & IVH	599	No	3	EML	25	75	DC, R SDH	H	4	VS
13	70	F	RTA	Bilateral frontal, parietal, corpus callosum & midbrain contusions. tSAH & IVH	23	Yes	3	2	21	34			1	GR
14	27	M	RTA	Bifrontal contusions. R frontal SDH	52	No	7	NEML	16	25			4	GR

M, male; F, female; RTA, road traffic accident, DAI, diffuse axonal injury; tSAH, traumatic subarachnoid haemorrhage, SDH, subdural haemorrhage; IVH, intraventricular haemorrhage; EDH, extradural haemorrhage, GCS, Glasgow coma score; NEML, non evacuated mass lesion; EML, evacuated mass lesion; EVD external ventricular drain; DC, decompressive craniectomy; GOS, Glasgow Outcome Score at 6 – 12 months post injury; MD, moderate disability; VS, vegetative state; SD, severe disability, GR, good recovery; NA, not available.

**Table 4:** Region of interest data within white matter regions in healthy volunteers and normoxic patients.

	*FA*			*AD*			*RD*			*MD*		
	Control	Normoxia	p value	Control	Normoxia	p value	Control	Normoxia	p value	Control	Normoxia	p value
ACC	0.63677 ± 0.12020	0.49887 ± 0.20845	0.0095	0.00158 ± 0.00011	0.00136 ± 0.00053	0.034	0.00048 ± 0.00013	0.00050 ± 0.00023	0.6457	0.00085 ± 0.0007	0.00080 ± 0.00030	0.3749
BCC	0.58257 ± 0.18841	0.33346 ± 0.20998	**0.0004**	0.00166 ± 0.00012	0.00117 ± 0.00055	**<0.0001**	0.00064 ± 0.00028	0.00062 ± 0.00027	0.8352	0.00097 ± 0.00019	0.00081 ± 0.00034	0.0438
PCC	0.70273 ± 0.08119	0.57710 ± 0.13015	**0.0004**	0.00177 ± 0.00022	0.00139 ± 0.00029	**<0.0001**	0.00056 ± 0.00035	0.00045 ± 0.00015	0.2763	0.00095 ± 0.00027	0.00078 ± 0.00014	0.0280
ATR left	0.40868 ± 0.01945	0.35482 ± 0.07519	**0.0004**	0.00119 ± 0.00003	0.00105 ± 0.00018	**<0.0001**	0.00064 ± 0.00004	0.00057 ± 0.00009	**0.0003**	0.00083 ± 0.0004	0.00074 ± 0.00011	**0.0002**
ATR right	0.36912 ± 0.01944	0.29968 ± 0.09557	**0.0003**	0.00127 ± 0.00004	0.00107 ± 0.00035	0.0035	0.00076 ± 0.00005	0.00065 ± 0.00023	0.0139	0.00093 ± 0.00005	0.00081 ± 0.00025	0.0115
SLF left	0.34637 ± 0.01184	0.31853 ± 0.06529	0.0229	0.00113 ± 0.00003	0.00106 ± 0.00022	0.0638	0.00070 ± 0.00003	0.00064 ± 0.00014	0.0255	0.00084 ± 0.00003	0.00078 ± 0.00017	0.0380
SLF right	0.37167 ± 0.01266	0.32277 ± 0.05725	**<0.0001**	0.00114 ± 0.00002	0.00104 ± 0.00017	**0.0014**	0.00067 ± 0.00002	0.00061 ± 0.00010	**0.0017**	0.00083 ± 0.00002	0.00076 ± 0.00012	**0.0020**
ILF left	0.38820 ± 0.01635	0.32670 ± 0.09123	**0.0005**	0.00117 ± 0.00002	0.00101 ± 0.00028	0.0029	0.00064 ± 0.00003	0.00056 ± 0.00016	0.0061	0.00081 ± 0.00002	0.00072 ± 0.00019	0.0059
ILF right	0.41278 ± 0.01966	0.31476 ± 0.13090	**0.0001**	0.00125 ± 0.00004	0.00099 ± 0.00041	**0.0008**	0.00066 ± 0.00004	0.00053 ± 0.00022	0.0026	0.00085 ± 0.00003	0.00069 ± 0.00028	0.0025
C left	0.30335 ± 0.03759	0.30404 ± 0.04193	**0.9562**	0.00118 ± 0.00005	0.00113 ± 0.00021	0.2290	0.00075 ± 0.00006	0.00070 ± 0.00015	0.1165	0.00089 ± 0.00005	0.00085 ± 0.00017	0.1613
C right	0.30259 ± 0.05510	0.26216 ± 0.07964	0.0573	0.00131 ± 0.00008	0.00108 ± 0.00033	**0.0006**	0.00086 ± 0.00012	0.00070 ± 0.00024	0.0040	0.00101 ± 0.00010	0.00084 ± 0.00027	0.0029
UF left	0.39960 ± 0.01869	0.28661 ± 0.11856	**<0.0001**	0.00117 ± 0.00003	0.00091 ± 0.00037	**0.0002**	0.00063 ± 0.00003	0.00051 ± 0.00020	**0.0014**	0.00081 ± 0.00002	0.00066 ± 0.00023	**0.0006**
UF right	0.37650 ± 0.02579	0.24896 ± 0.13432	**<0.0001**	0.00127 ± 0.00005	0.00090 ± 0.00049	**0.0001**	0.00073 ± 0.00006	0.00053 ± 0.00029	**0.0005**	0.00091 ± 0.00005	0.00069 ± 0.00032	**0.0004**
CT left	0.48492 ± 0.01647	0.44921 ± 0.05182	**0.0009**	0.00128 ± 0.00003	0.00118 ± 0.00012	**<0.0001**	0.00063 ± 0.00004	0.00058 ± 0.00006	**0.0011**	0.00084 ± 0.00004	0.00078 ± 0.00007	**0.0002**
CT right	0.48655 ± 0.01715	0.43681 ± 0.06783	**0.0003**	0.00126 ± 0.00003	0.00113 ± 0.00015	**<0.0001**	0.00060 ± 0.00004	0.00053 ± 0.00007	**<0.0001**	0.00082 ± 0.00004	0.00073 ± 0.00009	**<0.0001**
F Mi	0.38713 ± 0.01928	0.29800 ± 0.11998	**0.0002**	0.00126 ± 0.00004	0.00105 ± 0.00043	0.0093	0.00071 ± 0.00004	0.00061 ± 0.00026	0.0413	0.00089 ± 0.00003	0.00077 ± 0.00029	0.0266
F Ma	0.41169 ± 0.03333	0.37233 ± 0.05808	0.0056	0.00134 ± 0.00008	0.00120 ± 0.00019	**0.0005**	0.00072 ± 0.00008	0.00064 ± 0.00011	0.0137	0.00093 ± 0.00008	0.00083 ± 0.00013	0.0028
VM	0.56403 ± 0.06007	0.51104 ± 0.17386	0.1515	0.00139 ± 0.00015	0.00109 ± 0.00036	**0.0006**	0.00050 ± 0.00011	0.00040 ± 0.00015	0.0104	0.00080 ± 0.00012	0.00064 ± 0.00021	0.0029
DM	0.53050 ± 0.04129	0.45870 ± 0.09509	**0.0014**	0.00125 ± 0.00007	0.00116 ± 0.00021	0.0341	0.00054 ± 0.00006	0.00055 ± 0.00012	0.6100	0.00077 ± 0.00005	0.00075 ± 0.00014	0.4959
CP left	0.50314 ± 0.02263	0.51864 ± 0.03881	0.1092	0.00113 ± 0.00006	0.00113 ± 0.00005	0.8293	0.00048 ± 0.00003	0.00047 ± 0.00004	0.3588	0.00070 ± 0.00004	0.00069 ± 0.00004	0.6371
CP right	0.52636 ± 0.01760	0.52704 ± 0.02997	0.9257	0.00114 ± 0.00004	0.00113 ± 0.00006	0.8368	0.00046 ± 0.00002	0.00046 ± 0.00004	0.7512	0.00068 ± 0.00002	0.00069 ± 0.00004	0.8190
P left	0.53110 ± 0.02903	0.52361 ± 0.06831	0.6193	0.00129 ± 0.00008	0.00121 ± 0.00018	0.0567	0.00056 ± 0.00009	0.00052 ± 0.00011	0.1752	0.00079 ± 0.00007	0.00075 ± 0.00013	0.1399
P right	0.52891 ± 0.03832	0.51575 ± 0.05788	0.3833	0.00128 ± 0.00007	0.00118 ± 0.00012	**0.0009**	0.00055 ± 0.00007	0.00051 ± 0.00007	0.1515	0.00079 ± 0.00007	0.00073 ± 0.00008	0.0212
Mean	0.45335 ± 0.11535	0.39476 ± 0.14502		0.00129 ± 0.00018	0.00112 ± 0.00032		0.00063 ± 0.00015	0.00056 ± 0.00018		0.00085 ± 0.00011	0.00075 ± 0.00020	

Data are mean ± standard deviation using the atlas regions of interest applied in normalised space for fractional anisotropy (FA), mean diffusivity (MD) mm^2^/second, axial (AD) mm^2^/second and radial diffusivity (RD) mm^2^/second for 32 healthy volunteers and 14 patients with head injury. Anterior corpus callosum (ACC), body corpus callosum (BCC), posterior corpus callosum (PCC), anterior thalamic radiation (ATR), superior longitudinal fasciculus (SLF), inferior longitudinal fasciculus (ILF), Cingulum (C), uncinate fasciculus (UF), corticospinal tract (CT), forceps minor (F Mi), forceps major (F Ma), ventral midbrain (VM), dorsal midbrain (DM), cerebral peduncle (CP), pons (P). For the comparison between normoxic patients and healthy controls unpaired t-tests with Bonferroni correction for multiple comparisons were utilised, and a p < 0.0022 was considered significant. Significant results are highlighted in bold.

**Table 5:** Region of interest data within mixed cortical and deep grey matter regions in healthy volunteers and normoxic patients.

	*FA*			*AD*			*RD*			*MD*		
	Control	Normoxia	p value	Control	Normoxia	p value	Control	Normoxia	p value	Control	Normoxia	p value
Caud left	0.26092 ± 0.05327	0.29957 ± 0.07987	0.0688	0.00137 ± 0.00042	0.00098 ± 0.00021	0.0027	0.00101 ± 0.00039	0.00060 ± 0.00014	**0.0005**	0.00112 ± 0.00041	0.00074 ± 0.00014	**0.0016**
Caud right	0.28511 ± 0.04115	0.22121 ± 0.08821	**0.0025**	0.00100 ± 0.00012	0.00103 ± 0.00054	0.8329	0.00066 ± 0.00010	0.00074 ± 0.00045	0.3801	0.00078 ± 0.00010	0.00085 ± 0.00046	0.4196
Thal left	0.34373 ± 0.01600	0.34940 ± 0.09018	0.7457	0.00105 ± 0.00003	0.00098 ± 0.00028	0.2294	0.00064 ± 0.00003	0.00056 ± 0.00019	0.0205	0.00078 ± 0.00003	0.00070 ± 0.00022	0.0755
Thal right	0.34814 ± 0.01676	0.33398 ± 0.09947	0.4630	0.00104 ± 0.00003	0.00111 ± 0.00038	0.3440	0.00063 ± 0.00002	0.00067 ± 0.00028	0.4024	0.00077 ± 0.00002	0.00082 ± 0.00031	0.3799
H left	0.28230 ± 0.01721	0.27675 ± 0.04647	0.5754	0.00131 ± 0.00006	0.00119 ± 0.00027	0.0278	0.00089 ± 0.00006	0.00078 ± 0.00020	0.0120	0.00103 ± 0.00006	0.00092 ± 0.00023	0.0221
H right	0.28868 ± 0.01590	0.24806 ± 0.07484	0.0084	0.00143 ± 0.00006	0.00114 ± 0.00040	**0.0005**	0.00096 ± 0.00007	0.00075 ± 0.00028	**0.0003**	0.00112 ± 0.00006	0.00090 ± 0.00031	**0.0008**
F left	0.24658 ± 0.01024	0.23134 ± 0.04662	0.1028	0.00124 ± 0.00006	0.00116 ± 0.00024	0.1240	0.00091 ± 0.00005	0.00084 ± 0.00018	0.0605	0.00102 ± 0.00005	0.00095 ± 0.00020	0.0974
F right	0.24100 ± 0.00749	0.20716 ± 0.06476	0.0086	0.00126 ± 0.00005	0.00111 ± 0.00036	0.0287	0.00094 ± 0.00005	0.00081 ± 0.00028	0.0166	0.00105 ± 0.00005	0.00092 ± 0.00029	0.0267
P left	0.26126 ± 0.01020	0.25815 ± 0.02866	0.6087	0.00125 ± 0.00007	0.00114 ± 0.00020	0.0093	0.00090 ± 0.00006	0.00079 ± 0.00017	**0.0020**	0.00102 ± 0.00007	0.00091 ± 0.00018	0.0047
P right	0.26039 ± 0.00906	0.25118 ± 0.03396	0.1827	0.00127 ± 0.00005	0.00117 ± 0.00015	**0.0015**	0.00092 ± 0.00005	0.00082 ± 0.00012	**0.0001**	0.00104 ± 0.00005	0.00094 ± 0.00012	**0.0005**
Temp left	0.24581 ± 0.01440	0.20667 ± 0.08940	0.0277	0.00112 ± 0.00005	0.00093 ± 0.00042	0.0173	0.00079 ± 0.00004	0.00064 ± 0.00030	0.0100	0.00090 ± 0.00004	0.00075 ± 0.00033	0.0191
Temp right	0.25492 ± 0.01031	0.19311 ± 0.09262	**0.0011**	0.00120 ± 0.00003	0.00089 ± 0.00043	**0.0005**	0.00084 ± 0.00003	0.00062 ± 0.00030	**0.0002**	0.00096 ± 0.00003	0.00072 ± 0.00034	**0.0007**
O left	0.24578 ± 0.01287	0.25494 ± 0.01641	0.0544	0.00117 ± 0.00005	0.00108 ± 0.00013	**0.0029**	0.00084 ± 0.00005	0.00075 ± 0.00011	**0.0002**	0.00095 ± 0.00005	0.00086 ± 0.00011	**0.0007**
O right	0.23925 ± 0.01151	0.23656 ± 0.07214	0.8468	0.00120 ± 0.00006	0.00103 ± 0.00030	0.0042	0.00088 ± 0.00005	0.00072 ± 0.00020	**<0.0001**	0.00099 ± 0.00005	0.00082 ± 0.00023	**0.0008**
Cereb left	0.23434 ± 0.01828	0.22973 ± 0.04483	0.6374	0.00104 ± 0.00007	0.00097 ± 0.00013	0.0419	0.00074 ± 0.00007	0.00069 ± 0.00010	0.0432	0.00084 ± 0.00007	0.00078 ± 0.00011	0.0477
Cereb right	0.22928 ± 0.01782	0.22957 ± 0.02782	0.9674	0.00103 ± 0.00006	0.00099 ± 0.00008	0.0848	0.00074 ± 0.00006	0.00070 ± 0.00007	0.0920	0.00084 ± 0.00006	0.00080 ± 0.00008	0.0919
Mean	0.26672 ± 0.04034	0.25173 ± 0.07812		0.00119 ± 0.00017	0.00106 ± 0.00031		0.00083 ± 0.00016	0.00072 ± 0.00024		0.00095 ± 0.00016	0.00084 ± 0.00025	

Data are mean ± standard deviation using the atlas regions of interest applied in normalised space for fractional anisotropy (FA), mean diffusivity (MD) mm^2^/second, axial (AD) mm^2^/second and radial diffusivity (RD) mm^2^/second for 32 healthy volunteers and 14 patients with head injury. Caudate (Caud), thalamus (Thal), hippocampus (H), frontal (F), parietal (P), temporal (Temp), occipital (O), cerebellum (Cereb). For the comparison between normoxic patients and healthy controls unpaired t-tests with Bonferroni correction for multiple comparisons were utilised, and a p < 0.0031 was considered significant. Significant results are highlighted in bold.

### Impact of hyperoxia in patients

The ROI data in patients at normoxia and following hyperoxia for white matter and mixed cortical and deep grey matter are shown in Tables 6 and 7 respectively. These demonstrate that there were no changes in AD and MD. Within white matter FA was lower and RD higher within the left uncinate fasciculus (p < 0.05, paired t tests with Bonferroni correction). Within mixed cortical and deep grey matter FA was significantly lower following hyperoxia within the right caudate and occipital regions (p < 0.05, paired t tests with Bonferroni correction).

**Table 6:** Region of interest data within white matter regions in normoxic and hyperoxic patients.

	*FA*			*AD*			*RD*			*MD*		
	Normoxia	Hyperoxia	p value	Normoxia	Hyperoxia	p value	Normoxia	Hyperoxia	p value	Normoxia	Hyperoxia	p value
ACC	0.49887 ± 0.20845	0.46601 ± 0.19273	0.0334	0.00136 ± 0.00053	0.00131 ± 0.00052	0.0529	0.00050 ± 0.00023	0.00050 ± 0.00024	0.8849	0.00080 ± 0.00030	0.00077 ± 0.00031	0.0554
BCC	0.33346 ± 0.20998	0.33884 ± 0.21408	0.4982	0.00117 ± 0.00055	0.00116 ± 0.00054	0.2799	0.00062 ± 0.00027	0.00060 ± 0.00025	0.1193	0.00081 ± 0.00034	0.00079 ± 0.00032	0.0456
PCC	0.57710 ± 0.13015	0.57336 ± 0.12501	0.7631	0.00139 ± 0.00029	0.00137 ± 0.00032	0.7383	0.00045 ± 0.00015	0.00045 ± 0.00015	0.5701	0.00078 ± 0.00014	0.00076 ± 0.00018	0.3469
ATR left	0.35482 ± 0.07519	0.33935 ± 0.06744	0.0220	0.00105 ± 0.00018	0.00103 ± 0.00018	0.0114	0.00057 ± 0.00009	0.00056 ± 0.00010	0.2084	0.00074 ± 0.00011	0.00072 ± 0.00013	0.0613
ATR right	0.29968 ± 0.09557	0.28401 ± 0.09722	0.0061	0.00107 ± 0.00035	0.00107 ± 0.00034	0.4104	0.00065 ± 0.00023	0.00065 ± 0.00022	0.4066	0.00081 ± 0.00025	0.00079 ± 0.00026	0.0466
SLF left	0.31853 ± 0.06529	0.31750 ± 0.06472	0.8144	0.00106 ± 0.00022	0.00106 ± 0.00022	0.9032	0.00064 ± 0.00014	0.00064 ± 0.00014	0.9765	0.00078 ± 0.00017	0.00078 ± 0.00017	0.8687
SLF right	0.32277 ± 0.05725	0.32151 ± 0.05791	0.7934	0.00104 ± 0.00017	0.00104 ± 0.00018	0.9763	0.00061 ± 0.00010	0.00061 ± 0.00010	0.6550	0.00076 ± 0.00012	0.00075 ± 0.00013	0.6819
ILF left	0.32670 ± 0.09123	0.32196 ± 0.08965	0.0556	0.00101 ± 0.00028	0.00101 ± 0.00028	0.3297	0.00056 ± 0.00016	0.00056 ± 0.00016	0.9433	0.00072 ± 0.00019	0.00071 ± 0.00020	0.1586
ILF right	0.31476 ± 0.13090	0.30828 ± 0.12736	0.0448	0.00099 ± 0.00041	0.00099 ± 0.00041	0.7629	0.00053 ± 0.00022	0.00053 ± 0.00022	0.1948	0.00069 ± 0.00028	0.00068 ± 0.00028	0.2155
C left	0.30404 ± 0.04193	0.29809 ± 0.03727	0.2383	0.00113 ± 0.00021	0.00110 ± 0.00018	0.1035	0.00070 ± 0.00015	0.00068 ± 0.00015	0.0484	0.00085 ± 0.00017	0.00082 ± 0.00016	0.0695
C right	0.26216 ± 0.07964	0.23905 ± 0.07162	0.0130	0.00108 ± 0.00033	0.00104 ± 0.00033	0.0988	0.00070 ± 0.00024	0.00068 ± 0.00024	0.2510	0.00084 ± 0.00027	0.00080 ± 0.00027	0.0910
UF left	0.28661 ± 0.11856	0.27719 ± 0.11528	**0.0011**	0.00091 ± 0.00037	0.00091 ± 0.00037	0.6681	0.00051 ± 0.00020	0.00052 ± 0.00020	**0.0021**	0.00066 ± 0.00023	0.00065 ± 0.00026	0.3428
UF right	0.24896 ± 0.13432	0.24308 ± 0.13207	0.0097	0.00090 ± 0.00049	0.00090 ± 0.00049	0.7409	0.00053 ± 0.00029	0.00053 ± 0.00030	0.3017	0.00069 ± 0.00032	0.00065 ± 0.00036	0.1331
CT left	0.44921 ± 0.05182	0.44727 ± 0.04226	0.8566	0.00118 ± 0.00012	0.00119 ± 0.00009	0.5719	0.00058 ± 0.00006	0.00059 ± 0.00004	0.2257	0.00078 ± 0.00007	0.00079 ± 0.00005	0.3778
CT right	0.43681 ± 0.06783	0.43413 ± 0.06456	0.8495	0.00113 ± 0.00015	0.00114 ± 0.00014	0.6354	0.00053 ± 0.00007	0.00054 ± 0.00006	0.2061	0.00073 ± 0.00009	0.00074 ± 0.00009	0.5114
F Mi	0.29800 ± 0.11998	0.28593 ± 0.11483	0.0120	0.00105 ± 0.00043	0.00105 ± 0.00043	0.8826	0.00061 ± 0.00026	0.00062 ± 0.00025	0.0090	0.00077 ± 0.00029	0.00077 ± 0.00031	0.5523
F Ma	0.37233 ± 0.05808	0.36734 ± 0.05747	0.0326	0.00120 ± 0.00019	0.00119 ± 0.00019	0.7544	0.00064 ± 0.00011	0.00064 ± 0.00011	0.5321	0.00083 ± 0.00013	0.00083 ± 0.00013	0.9731
VM	0.51104 ± 0.17386	0.41313 ± 0.15136	0.0208	0.00109 ± 0.00036	0.00098 ± 0.00036	0.0344	0.00040 ± 0.00015	0.00036 ± 0.00014	0.0326	0.00064 ± 0.00021	0.00057 ± 0.00021	0.0213
DM	0.45870 ± 0.09509	0.45470 ± 0.09622	0.6912	0.00116 ± 0.00021	0.00115 ± 0.00022	0.5138	0.00055 ± 0.00012	0.00054 ± 0.00012	0.3076	0.00075 ± 0.00014	0.00074 ± 0.00015	0.3755
CP left	0.51864 ± 0.03881	0.50183 ± 0.03786	0.2359	0.00113 ± 0.00005	0.00110 ± 0.00008	0.2290	0.00047 ± 0.00004	0.00046 ± 0.00005	0.2358	0.00069 ± 0.00004	0.00067 ± 0.00005	0.1441
CP right	0.52704 ± 0.02997	0.51477 ± 0.03095	0.1299	0.00113 ± 0.00006	0.00112 ± 0.00006	0.4128	0.00046 ± 0.00004	0.00046 ± 0.00005	0.9786	0.00069 ± 0.00004	0.00068 ± 0.00005	0.5297
P left	0.52361 ± 0.06831	0.49732 ± 0.08016	0.1299	0.00121 ± 0.00018	0.00118 ± 0.00020	0.0358	0.00052 ± 0.00011	0.00050 ± 0.00012	0.1257	0.00075 ± 0.00013	0.00073 ± 0.00014	0.0394
P right	0.51575 ± 0.05788	0.48313 ± 0.07295	0.0630	0.00118 ± 0.00012	0.00116 ± 0.00014	0.2579	0.00051 ± 0.00007	0.00051 ± 0.00009	0.9642	0.00073 ± 0.00008	0.00073 ± 0.00010	0.6623
Mean	0.39476 ± 0.14502	0.38033 ± 0.13861		0.00112 ± 0.00032	0.00110 ± 0.00031		0.00056 ± 0.00018	0.00055 ± 0.00018		0.00075 ± 0.00020	0.00074 ± 0.00021	

Data are mean ± standard deviation using the atlas regions of interest applied in normalised space for fractional anisotropy (FA), mean diffusivity (MD) mm^2^/second, axial (AD) mm^2^/second and radial diffusivity (RD) mm^2^/second for 14 patients with head injury. Anterior corpus callosum (ACC), body corpus callosum (BCC), posterior corpus callosum (PCC), anterior thalamic radiation (ATR), superior longitudinal fasciculus (SLF), inferior longitudinal fasciculus (ILF), Cingulum (C), uncinate fasciculus (UF), corticospinal tract (CT), forceps minor (F Mi), forceps major (F Ma), ventral midbrain (VM), dorsal midbrain (DM), cerebral peduncle (CP), pons (P). For the comparison between normoxic and hyperoxic patients paired t-tests with Bonferroni correction for multiple comparisons were utilised, and a p < 0.0022 was considered significant. Significant results are highlighted in bold.

**Table 7:** Region of interest data within mixed cortical and deep grey matter regions in normoxic and hyperoxic patients.

	FA			AD			RD			MD		
	Normoxia	Hyperoxia	p value	Normoxia	Hyperoxia	p value	Normoxia	Hyperoxia	p value	Normoxia	Hyperoxia	p value
Caud left	0.26092 ± 0.05327	0.27767 ± 0.07919	0.0545	0.00137 ± 0.00042	0.00097 ± 0.00022	0.2827	0.00101 ± 0.00039	0.00061 ± 0.00013	0.8614	0.00112 ± 0.00041	0.00073 ± 0.00016	0.3045
Caud right	0.28511 ± 0.04115	0.19793 ± 0.08400	**0.0010**	0.00100 ± 0.00012	0.00103 ± 0.00054	0.7154	0.00066 ± 0.00010	0.00076 ± 0.00044	0.3644	0.00078 ± 0.00010	0.00085 ± 0.00047	0.9001
Thal left	0.34373 ± 0.01600	0.34006 ± 0.09364	0.3427	0.00105 ± 0.00003	0.00096 ± 0.00025	0.2320	0.00064 ± 0.00003	0.00054 ± 0.00016	0.3468	0.00078 ± 0.00003	0.00068 ± 0.00019	0.2857
Thal right	0.34814 ± 0.01676	0.31502 ± 0.09599	0.1183	0.00104 ± 0.00003	0.00107 ± 0.00034	0.2343	0.00063 ± 0.00002	0.00065 ± 0.00023	0.5554	0.00077 ± 0.00002	0.00079 ± 0.00026	0.3623
H left	0.28230 ± 0.01721	0.26040 ± 0.04050	0.0268	0.00131 ± 0.00006	0.00118 ± 0.00027	0.2343	0.00089 ± 0.00006	0.00079 ± 0.00020	0.1988	0.00103 ± 0.00006	0.00092 ± 0.00022	0.7549
H right	0.28868 ± 0.01590	0.22844 ± 0.07136	0.0244	0.00143 ± 0.00006	0.00115 ± 0.00039	0.5317	0.00096 ± 0.00007	0.00077 ± 0.00028	0.1794	0.00112 ± 0.00006	0.00090 ± 0.00031	0.8673
F left	0.24658 ± 0.01024	0.22486 ± 0.02626	0.3944	0.00124 ± 0.00006	0.00117 ± 0.00021	0.7043	0.00091 ± 0.00005	0.00085 ± 0.00017	0.1921	0.00102 ± 0.00005	0.00096 ± 0.00019	0.7320
F right	0.24100 ± 0.00749	0.19822 ± 0.05414	0.1786	0.00126 ± 0.00005	0.00111 ± 0.00033	0.7611	0.00094 ± 0.00005	0.00082 ± 0.00026	0.2848	0.00105 ± 0.00005	0.00092 ± 0.00028	0.7473
P left	0.26126 ± 0.01020	0.24623 ± 0.02936	0.1439	0.00125 ± 0.00007	0.00114 ± 0.00020	0.9095	0.00090 ± 0.00006	0.00080 ± 0.00016	0.3040	0.00102 ± 0.00007	0.00091 ± 0.00017	0.5460
P right	0.26039 ± 0.00906	0.24493 ± 0.03334	0.2826	0.00127 ± 0.00005	0.00118 ± 0.00013	0.9294	0.00092 ± 0.00005	0.00084 ± 0.00010	0.2873	0.00104 ± 0.00005	0.00095 ± 0.00011	0.5020
Temp left	0.24581 ± 0.01440	0.18931 ± 0.08033	0.0098	0.00112 ± 0.00005	0.00091 ± 0.00041	0.7466	0.00079 ± 0.00004	0.00065 ± 0.00029	0.6335	0.00090 ± 0.00004	0.00074 ± 0.00033	0.2284
Temp right	0.25492 ± 0.01031	0.17901 ± 0.08160	0.0203	0.00120 ± 0.00003	0.00088 ± 0.00041	0.3056	0.00084 ± 0.00003	0.00063 ± 0.00030	0.5390	0.00096 ± 0.00003	0.00071 ± 0.00034	0.1902
O left	0.24578 ± 0.01287	0.24138 ± 0.01921	0.0185	0.00117 ± 0.00005	0.00107 ± 0.00012	0.3962	0.00084 ± 0.00005	0.00075 ± 0.00011	0.3149	0.00095 ± 0.00005	0.00086 ± 0.00011	0.8351
O right	0.23925 ± 0.01151	0.21773 ± 0.06625	**0.0025**	0.00120 ± 0.00006	0.00101 ± 0.00029	0.5054	0.00088 ± 0.00005	0.00072 ± 0.00020	0.9072	0.00099 ± 0.00005	0.00082 ± 0.00023	0.5505
Cereb left	0.23434 ± 0.01828	0.22161 ± 0.03535	0.2294	0.00104 ± 0.00007	0.00098 ± 0.00011	0.1807	0.00074 ± 0.00007	0.00070 ± 0.00009	0.0878	0.00084 ± 0.00007	0.00079 ± 0.00010	0.1024
Cereb right	0.22928 ± 0.01782	0.22366 ± 0.02530	0.1595	0.00103 ± 0.00006	0.00099 ± 0.00011	0.2355	0.00074 ± 0.00006	0.00071 ± 0.00007	0.0806	0.00084 ± 0.00006	0.00080 ± 0.00007	0.1561
Mean	0.26672 ± 0.04034	0.23795 ± 0.07417		0.00119 ± 0.00017	0.00105 ± 0.00030		0.00083 ± 0.00016	0.00072 ± 0.00023		0.00095 ± 0.00016	0.00083 ± 0.00025	

Data are mean ± standard deviation using the atlas regions of interest applied in normalised space for fractional anisotropy (FA), mean diffusivity (MD) mm^2^/second, axial (AD) mm^2^/second and radial diffusivity (RD) mm^2^/second for 14 patients with head injury. Caudate (Caud), thalamus (Thal), hippocampus (H), frontal (F), parietal (P), temporal (Temp), occipital (O), cerebellum (Cereb). For the comparison between normoxic and hyperoxic patients paired t-tests with Bonferroni correction for multiple comparisons were utilised, and a p < 0.0031 was considered significant. Significant results are highlighted in bold.

**Figure 1:**
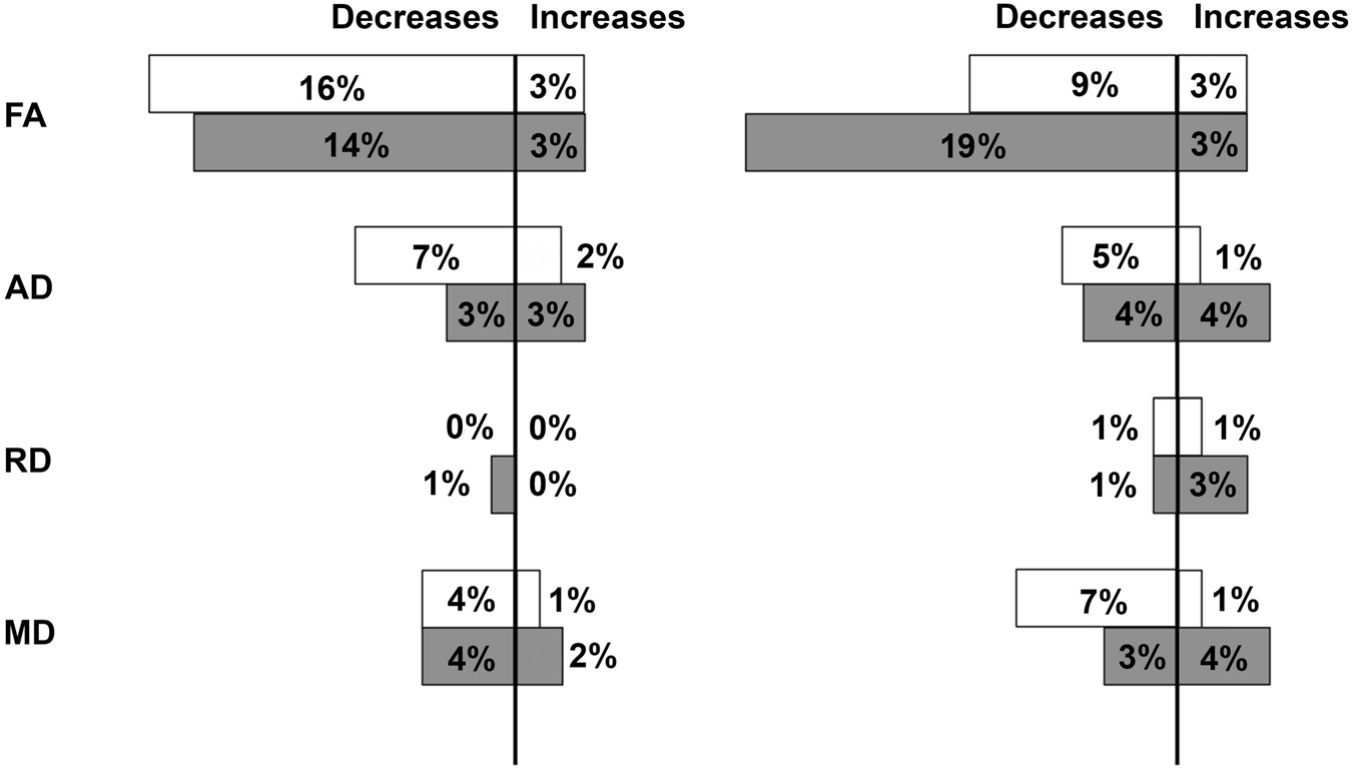
Impact of hyperoxia in patients. Fractional anisotropy (FA), axial diffusivity (AD), radial diffusivity (RD) and mean diffusivity (MD) within atlas regions of interest (ROI) applied in normalised space for 14 patients using “lesion free” brain by exclusion of lesion core and contusion tissue. Data displayed are the percentage number of white (white) and mixed cortical and deep grey matter (grey) ROIs showing a change greater than the overall population (left panel) and individual regional (right panel) 99% prediction interval (PI) for zero change. The total number of regions in this cohort was 320 and 223 for white matter and mixed cortical and deep grey matter respectively.

**Figure 2:**
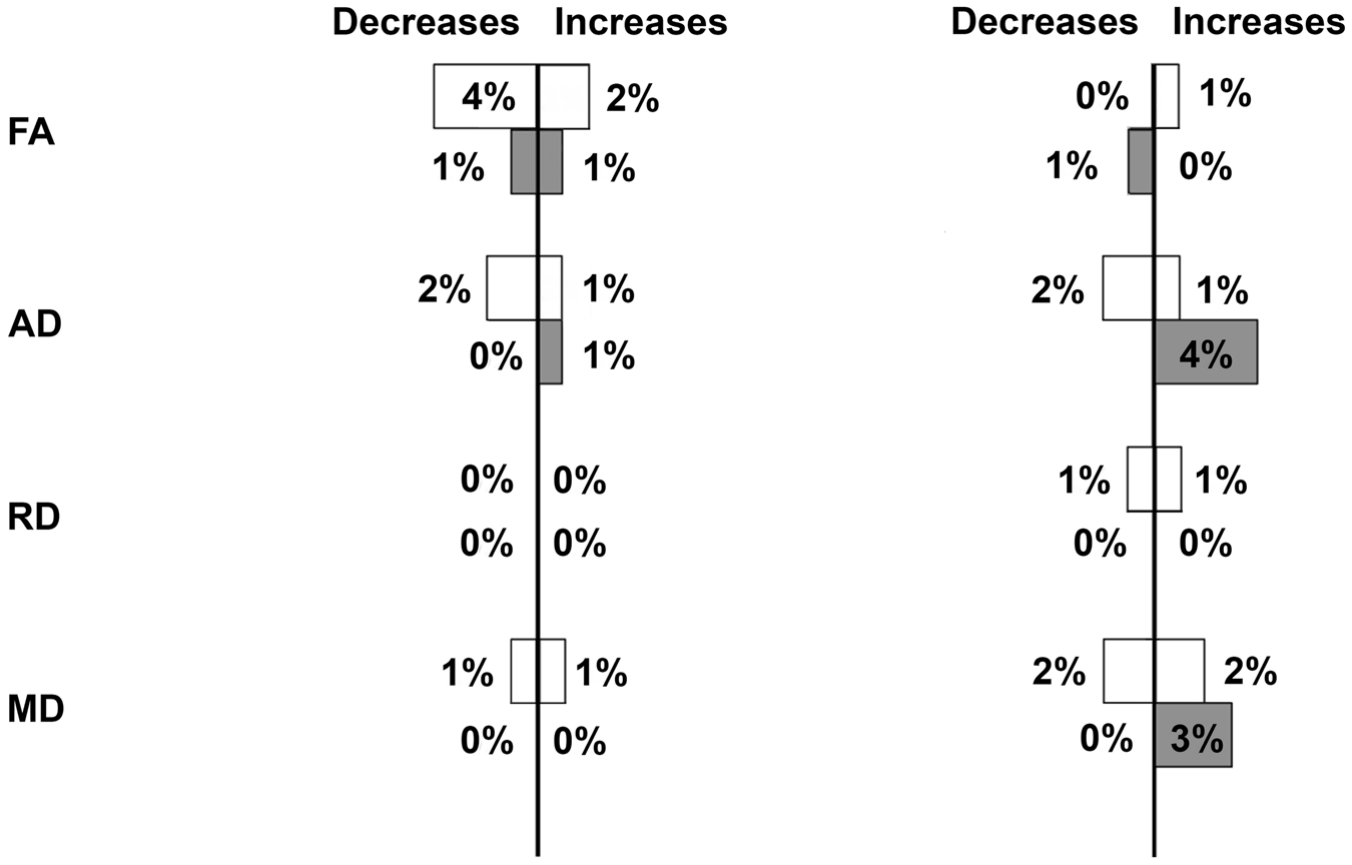
Impact of hyperoxia in healthy volunteers. Fractional anisotropy (FA), axial diffusivity (AD), radial diffusivity (RD) and mean diffusivity (MD) within atlas regions of interest (ROI) applied in normalised space for 6 healthy volunteers. Data displayed are the percentage number of white (white) and mixed cortical and deep grey matter (grey) ROIs showing a change greater than the overall population (left panel) and individual regional (right panel) 99% prediction interval (PI) for zero change. The total number of regions in this cohort was 138 and 96 for white matter and mixed cortical and deep grey matter respectively.

The percentage of white and mixed cortical and deep grey matter ROIs in patients and healthy volunteers exposed to hyperoxia showing a change in DTI parameters following NH that was greater than the overall population and regional 99% prediction intervals (PIs) for zero change are summarised in Figs 1 and 2 respectively. In healthy volunteers these changes are shown from baseline air to 100% oxygen. Using the overall population 99% PI significant decreases in FA were found within 16% of white matter ROIs from 9/14 patients and in 14% of mixed cortical and deep grey matter ROIs from 8/14 patients. Changes in the other DTI parameters were less frequent; some regions showed significant decreases in AD and MD while RD was generally unchanged. Supplementary Tables S1 and S2 provide a detailed list of which patients and regions showed significant change for white and mixed cortical and deep grey matter regions respectively. Decreases in FA were found across the whole brain in many different brain regions. Supplementary Tables S3 and S4 provide the equivalent data for the 6 healthy volunteers who underwent graded exposure to oxygen. The results using the individual ROI reproducibility data were similar and demonstrate significant FA decreases in 9% of white and 19% of mixed cortical and deep grey matter ROIs respectively (see Figs 1 and 2). Supplementary Tables S5 and S6 provide a detailed list of the regions showing significant change for white and mixed cortical and deep grey matter in patients, while S7 and S8 show the equivalent for healthy volunteers.

## Discussion

In this study we used DTI to examine whether an increase in the fraction of inspired oxygen had any beneficial effects within deep grey and mixed cortical, and white matter regions distant from visible contusions following TBI. Baseline patient data showed evidence of traumatic injury with lower MD and FA in several regions compared with healthy volunteers, consistent with cytotoxic oedema and axonal injury respectively. Exposure to a brief period of NH had no effect on healthy volunteers, and did not ameliorate these findings in patients with some regions showing further FA decreases following intervention. Using published reproducibility data from a historical cohort of 26 healthy volunteers we demonstrated that 16% of white matter and 14% of mixed cortical and deep grey matter regions in patients showed a reduction in FA more than the expected population 99% PI for zero change. The mechanistic basis for some of the DTI findings are unclear, but imply that a short period of NH has no beneficial impact within brain that appears normal using conventional structural imaging. To confirm these findings and investigate further will require a longer duration of hyperoxia with serial DTI and conventional MRI in comparison with clinical outcome.

Monitoring of focal tissue oxygen and brain metabolism using microdialysis has shown that hyperoxia can correct derangements^4,13,14^ and may be associated with improved outcome^1,15^. Further, a ^15^O PET study suggested that improvements in metabolism with hyperoxia may be particularly relevant within brain regions with physiology consistent with the greatest risk of infarction^4^. We have also used DTI to demonstrate contusion expansion within a rim of low MD consistent with cytotoxic oedema that surrounds a region of high MD (vasogenic oedema)^11^, and normobaric hyperoxia can increase MD values towards normal within this perilesional rim^5^. Both these imaging studies demonstrate how a short period of exposure to normobaric hyperoxia (~60 minutes) can result in potential benefit. Such findings suggest improvements in oxygen delivery that may relate to evidence of microvascular injury^16^ within the ‘traumatic penumbra’ and are consistent with post mortem studies showing microvascular occlusion and perivascular oedema associated with selective neuronal loss post TBI^17,18^. Increased brain oxygen levels may overcome diffusion barriers to oxygen delivery^5^ or improve mitochondrial function where low oxygen tension allows nitric oxide to competitively inhibit cytochrome oxidase^19^. Mitochondrial dysfunction has been shown in *ex vivo* clinical and experimental TBI studies^20^, and mitochondrial function can be preserved using hyperoxia^21^. Other studies demonstrate that hyperoxia has neuroprotective and anti-inflammatory effects within the injured brain^22^.

Whilst these changes are most evident within perilesional regions pathophysiological derangements are also evident in regions distant from visible injury based on conventional structural imaging^12,23,24^. Several PET studies have shown evidence of ischaemia and other metabolic derangements within brain that may initially appear structurally normal^16,23,25,26^ but ultimately demonstrates late atrophy, and is associated with poor outcome^27^. Further, benefit shown with normobaric hyperoxia in the ^15^O PET study by Nortje *et al*.^4^ within brain demonstrating physiology consistent with the greatest risk of infarction included normal appearing white matter. Studies using DTI are particularly relevant in this regard since evidence of cytotoxic oedema and traumatic axonal injury are often identified using this technique when conventional structural imaging appears normal^12,28^. Our findings were consistent with these data. Despite the exclusion of visible contusions and other areas of brain injury, the patient regional baseline data demonstrated significant DTI abnormalities consistent with cytotoxic oedema and axonal injury in comparison with healthy controls. Such regions were the focus of this study, and our expectation was that we may see amelioration of cytotoxic oedema and other DTI signal changes in brain distant from contusions following hyperoxia secondary to an improvement in oxygen delivery and/or mitochondrial function. It is important to acknowledge that any change must be sustained if it is to result in improved neuronal survival and better functional outcome for patients, but it is likely that this will require a much longer period of exposure to NH. However, we wished to demonstrate whether it was possible to use DTI as a biomarker of the trajectory of such injury or its recovery in the assessment of therapeutic interventions such as hyperoxia. Previous imaging studies have limited exposure to NH to one hour^4,5^, and have conducted repeat imaging within a single session in which changes in other physiological and patient related factors can be minimised. There are also concerns regarding excessive exposure to NH since it can result in atelectasis and pulmonary injury, increased oxidative stress and potential harm in critically ill patients. In this context a further preliminary study of the impact of NH on the injured brain was warranted.

Experimental and clinical ischaemia following middle cerebral artery occlusion results in early evidence of cytotoxic oedema with a reduction in MD^29^, and while AD and RD are typically reduced, it has been hypothesised that oligodendrite swelling can compress the axoplasm and result in a greater decrease in RD than AD within white matter^30^. This may explain why acute ischaemia can result in an initial increase in white matter FA if imaging is conducted within 4.5 hours of acute stroke^30,31^. Later, loss of cellular integrity results in large decreases in white matter FA^30^. These finding are relevant to ischaemic stroke, but were hyperoxia to improve oxygen delivery and attenuate cytotoxic oedema following TBI, MD should increase towards normal and, in theory, an increase in RD that was greater than AD could result in an initial *reduction* in white matter FA. While we did find evidence of low MD in TBI patients at baseline consistent with cytotoxic oedema we did not see evidence of an increase in MD towards normal within white or grey matter regions following exposure to hyperoxia. This suggests that the intervention was ineffective, or that a longer period of hyperoxia was needed to demonstrate any effect. In addition, the lack of evidence for a reversal of cytotoxic oedema cannot provide explanation for our finding of a reduction in FA within white or grey matter.

We exposed healthy volunteers to oxygen therapy since oxygen has a known paramagnetic effect and could have resulted in systematic changes to our DTI findings^32^. We saw no relationship between a step increase in administered oxygen and any of the DTI parameters. Healthy volunteers received oxygen via a venturi mask, in comparison with TBI patients who received fixed concentrations of inspired oxygen via a closed ventilatory circuit as they had been intubated and ventilated as part of routine clinical care. The Venturi mask provides a means of reliably titrating the FiO_2_ in spontaneously breathing subjects^33^, and while arterial blood gases were not monitored in healthy volunteers each step increase in delivered oxygen will have resulted in higher PaO_2_. Following 15 minutes of breathing 60% oxygen volunteers underwent ~45 minutes of imaging (DTI and whole brain proton spectroscopy) whilst continuing to breath 60% oxygen. Then, following an additional 15 minutes breathing 100% oxygen imaging was repeated for the last time. So, by the final DTI sequence subjects had been breathing an increased fraction of inspired oxygen for over 60 minutes. At this stage the PaO_2_ of the healthy volunteers would have been at least as high as that achieved in patients^5^.

In patients, we looked for regions where changes in DTI were greater than the 99% PI for zero change using published data from 26 historical healthy volunteers who underwent DTI on up to 4 occasions within two imaging sessions^34^. Both patients and volunteers underwent scanning within the Wolfson Brain Imaging Centre (WBIC) using the same scanner, software version and scanner sequences. Since patients underwent baseline and post intervention imaging during the same session the expected variability in patients, who were also sedated and paralysed during imaging, is likely to be at least as good as that found in awake spontaneously breathing healthy volunteers who underwent repeat DTI during two sessions separated by up to six months^34,35^.

Patients suffered a TBI and the presence of brain lesions will produce errors in spatial processing, particularly were non-linear algorithms are used to co-register and transform data to a standard template. The ROI template was eroded by a single voxel to limit problems resulting from co-registration, normalisation and partial volume errors. Visible areas of injury were manually delineated in native space, and subsequently, a normalised binary mask of the lesions was used to exclude this volume of brain tissue from the individualised standard ROI template of each patient. All registered datasets were reviewed to ensure that the spatial processing had not resulting in significant errors, and no subjects were excluded on this basis. While these concerns may lead to an over estimate of the difference between regional DTI values in patients compared to healthy volunteers, it is important to emphasise that the focus of this study was to compare change following NH within individual subjects during the same imaging session. There were no structural differences between the baseline and post NH datasets, and therefore, any small errors in registration and normalisation would have been replicated in both datasets. Patients were sedated, paralysed and ventilated throughout imaging sessions as part of routine care. This would have prevented movement artefact and helped optimise data collection, processing and subsequent analyses. Under these circumstances small changes within individual ROIs that relate to problems with spatial processing would be unlikely to introduce systematic errors between baseline and post NH intervention imaging within individual subjects. While it is possible that the DTI changes we found occurred purely by chance, we cannot ignore the fact that over 10% of all patient regions showed a fall in FA greater than the 99% PI for zero change following NH.

While the significance of a fall in FA following a brief exposure to hyperoxia is unknown it still represents some detectable and reversible change in the local tissue environment that did not occur in healthy volunteers exposed to a similar intervention. Given the concern regarding the use of hyperoxia it would be important to exclude the possibility, however small, that this could represent some early evidence of axonal injury within white matter resulting from oxidative stress. In chronic TBI a reduction of FA within white matter is consistent with axonal injury, with the extent of changes dependent on the time since ictus^12^. Interestingly, late cortical FA increases can also occur and may relate to scarring post mild TBI^36^. Clearly, it would be important to undertake serial MRI with DTI to understand how these DTI parameters evolve within both grey and white matter following exposure to longer periods of NH. At the very least these findings demonstrate how such measurements could be used to assess the impact of a longer duration of therapeutic NH and should be compared with evidence of late tissue fate based on structural MR and clinical outcome. Finally, since AD and RD are parameters that relate to the orientation of white matter fibres the small changes we found within mixed cortical and deep grey matter following hyperoxia are of little consequence.

Patients underwent imaging between days 1 – 9 post injury, and DTI changes may reflect different trajectories within individual subjects with resolution of oedema mixed with loss of tissue integrity within established lesions. Despite this concern, there was no significant interaction between FA changes following hyperoxia and the time since injury (p = 0.59, ANOVA). All patients in this cohort sustained TBI severe enough to require intensive care management of raised intracranial pressure, and had comparable imaging patterns of injury (Table 3). Despite this, outcome was variable and it is possible that the changes in DTI parameters seen may reflect individual variability within this small cohort. However, reductions in FA were seen in over half the patients and in the majority of brain regions with no clear relationship to injury type or eventual outcome. Nevertheless, definitive statements concerning the significance of DTI changes would require data from a larger cohort of patients showing evidence of sustained reductions in FA associated with poor functional outcome in comparison with a control arm before it could be concluded that they were indicative of axonal injury^37^. Sequential imaging could be used as a biomarker of the trajectory of such injury or its recovery in the assessment of therapeutic interventions such as hyperoxia^12^.

Previous clinical studies have suggested that the use of high partial pressures of oxygen may be beneficial^1,3^, but there may be a relatively narrow margin of safety^9^. We limited the maximum FiO_2_ in this interventional study to 0.8 to minimise direct side effects such as alveolar atelectasis and pulmonary injury. Clinical studies show little evidence of increased oxidative stress when therapy is applied in a controlled manner within the first three days post injury^3,5,38^. We show how changes in DTI can be detected in patients following NH based on reproducibility data from a historical group of healthy volunteers. The pathophysiological basis and significance of any fall in FA following exposure to NH remains unknown, particularly following such a brief intervention. Nevertheless, any potentially adverse effect should be considered and further studies should incorporate serial DTI to help determine how and when this intervention should be used within a precision medicine approach to optimise the beneficial impact on patient outcome. Such data could be invaluable in the design of any future clinical trial since studies to date do not provide definitive evidence of an improvement in clinical outcome^3^.

Prior TBI studies have suggested that an increase in the fraction of inspired oxygen can improve cerebral metabolism within perilesional and normal appearing white matter^4,39^, and using DTI, result in benefit within the rim of cytotoxic oedema found around brain contusions^5^. Using DTI we showed evidence of cytotoxic oedema and traumatic axonal injury distant from visible lesions with no improvement following the short-term administration of normobaric hyperoxia. To confirm these findings and investigate further will require a longer duration of hyperoxia with serial DTI and conventional MRI in comparison with clinical outcome.

## Methods

Ethical approval was obtained from the Cambridgeshire Research Ethics Committee (reference numbers 97/290 and 02/293), and assent from next-of-kin with later written informed consent, where appropriate, obtained in all cases in accordance with the Declaration of Helsinki.

### Subjects

#### Patients

Fourteen adult patients (12 males and 2 females) with median (range) age 33 (21 – 70) years with TBI were recruited from the Neurosciences Critical Care Unit (NCCU), Addenbrooke’s Hospital, Cambridge, UK between 2010 and 2012. Patients presented with median (range) post resuscitation Glasgow Coma Score (GCS) of 7 (3–14), but all subsequently had a GCS <8 requiring sedation and ventilation for control of intracranial pressure (ICP) (Table 3). Patients were recruited between day 1 and day 9 post injury and underwent imaging whilst sedated and ventilated. Patients with previous TBI, other neurological disease, or contraindication to magnetic resonance imaging (MRI) were excluded. All patients were managed by protocol driven care; which included sedation, paralysis and ventilation to ensure that intracranial pressure (ICP) <20 mmHg and cerebral perfusion pressure >60 mmHg were maintained^4^. Physiological stability was meticulously ensured during imaging through the titration of fluids and vasoactive agents and the presence of a critical care physician and nurse. Patients who received surgical intervention (CSF drainage or decompressive craniectomy) or second-tier medical therapies (barbiturate coma or moderate hypothermia (33–35 °C)) before imaging are specified in Table 3.

Based on previous imaging studies we acquired baseline DTI at a partial pressure of oxygen (PaO_2_) of approximately 75–90 mmHg (10–12 kPa) and then increased the FiO_2_ to a maximum of 0.8 in order to achieve a PaO_2_ of approximately 225–260 mmHg (30–35 kPa). Following a 60-minute period to allow the impact of higher PaO_2_ (and by inference, brain pO_2_) levels on cerebral metabolism, repeat DTI was obtained within the same imaging session without moving the patient.

#### Controls

A total of 32 healthy volunteers (19 females and 13 males) with a median (range) age of 34 (22–52) years underwent DTI breathing room air. Six of these volunteers were exposed to graded oxygen therapy (60% and 100% inspired oxygen) delivered via a venturi mask (Flexicare Medical Limited, Mid Glamorgan, Wales) and underwent repeat DTI within the same imaging session. Diffusion tensor imaging and whole brain proton spectroscopy were obtained at each level of inspired oxygen (21%, 60% and 100%) following an equilibration period of 15 minutes. The baseline data obtained breathing room air in all 32 subjects, and the graded oxygen therapy data in the six healthy volunteers, are presented in the results section of this manuscript. As part of a previously published study, 26 of these volunteers underwent DTI on up to 4 occasions within two imaging sessions separated by a maximum of six months. The reproducibility data from this historical cohort have been published, and are used in the subsequent analyses^34^.

### Imaging

All subjects were scanned using a 3 T Siemens Verio MRI scanner (Siemens AG, Erlangen, Germany) within the WBIC, University of Cambridge. During the study period there were no major changes or upgrades to the scanner or software. Sequences included a 3D T1-weighted magnetization prepared rapid gradient echo (MPRAGE), fluid attenuated inversion recovery (FLAIR), gradient echo (GE), susceptibility weighted (SWI), dual spin echo (proton density/T2-weighted) and whole brain proton spectroscopy (26 minutes). The DTI data were acquired over 13:50 minutes using 63 non-collinear directions, b = 1000 s/mm^2^ with one volume acquired without diffusion weighting (b = 0), echo time (TE) 106ms, repetition time (TR) 11700ms, 63 slices, field of view 192mm × 92mm, and 2 × 2 × 2mm^3^ isotropic voxels. All imaging was reviewed by a specialist clinical neuroradiologist.

#### Image processing

Fractional anisotropy, MD and AD maps were created using the Oxford Centre for functional MRI of the brain FSL Diffusion Toolbox^40^, while RD values were calculated as the mean of the second and third eigenvalues. To aid coregistration, the skull and extracranial soft tissue were stripped from the MPRAGE image using the Brain Extraction Tool of FSL^41^. The diffusion weighted data were normalized to the Montreal Neurological Institute 152 (MNI152) template using the non-linear vtkCISG normalized mutual information algorithm^42^. Using the same non-linear algorithm the T1 weighted images were coregistered to the MNI152 template and each subject’s b = 0 image subsequently coregistered to the individual T1 weighted image. The transformation matrix normalizing the MPRAGE was then applied to the b = 0 image. All coregistered and normalized images were visually checked to ensure that they were aligned.

### Region of interest analysis

**Figure 3:**
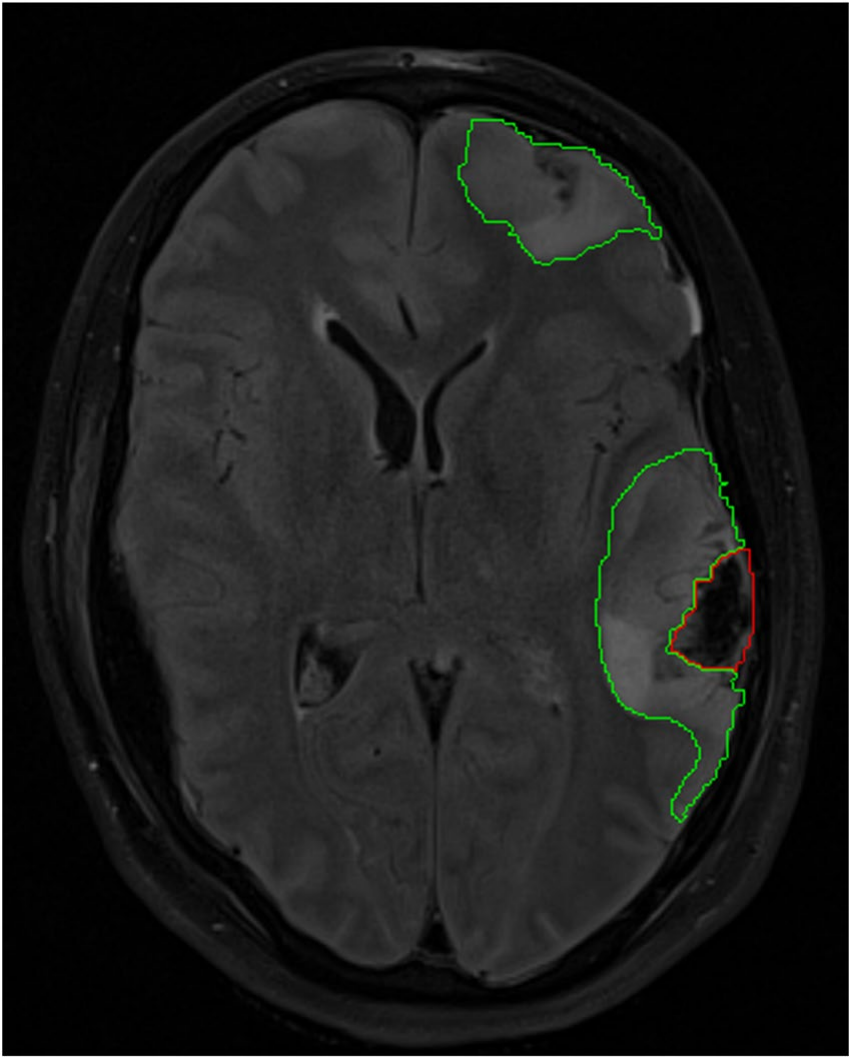
Lesion regions of Interest. Fluid attenuated inversion recovery (FLAIR) image from subject 8 with lesion core and contusion highlighted on a single axial slice.

Lesions were defined in native FLAIR space by a single author (JG) using patient FLAIR, MPRAGE, GE and SWI images. Lesion core was identified as a region of mixed signal intensity consistent with haemorrhage and necrotic tissue and contusion as an area of high signal on FLAIR (Fig. 3). The ROIs were drawn using Analyze 8.5 (Analyze Direct, Lenexa, KS, USA). FLAIR images were coregistered to T1 space using SPM8, and the coregistration matrix applied to the individual lesion ROIs.

**Figure 4:**
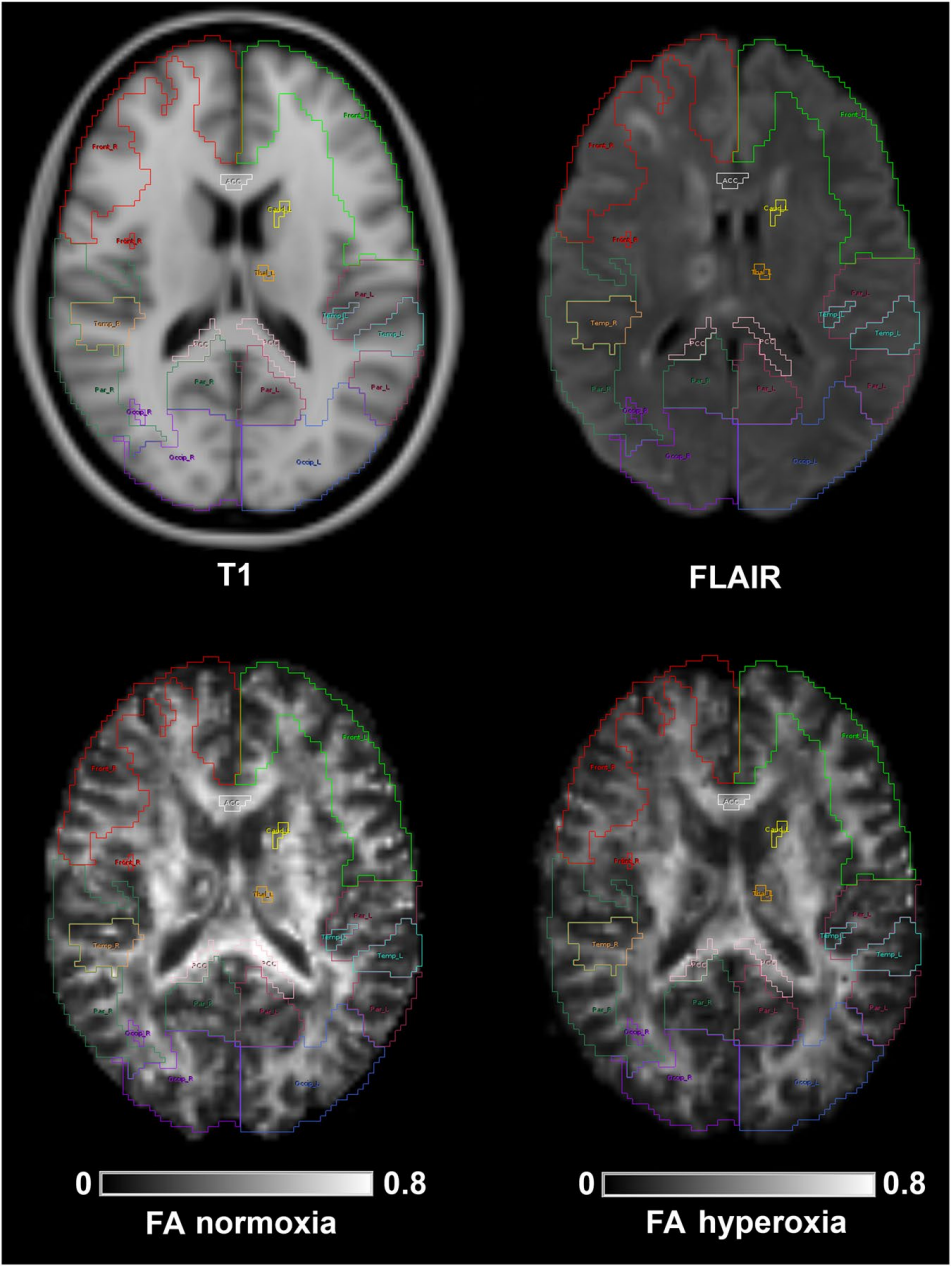
Individualised template regions of interest. Standard T1 weighted magnetic resonance image and patient fluid attenuated inversion recovery (FLAIR), with fractional anisotropy (FA) at baseline normoxia and following hyperoxia; all images are displayed in Montreal Neurological Institute 152 (MNI152) space. The region of interest (ROI) template for this subject (subject 1) has been individualised by the exclusion of lesion core and contusion tissue. On the FLAIR image slice shown lesions can be seen within the right frontal and temporal cortex, white matter, right caudate and right thalamus. On this axial slice regions shown include frontal left (Front_L), frontal right (Front_R), temporal left (Temp_L), temporal right (Temp_R), parietal left (Par_L), parietal right (Par_R), occipital left (Occip_L), occipital right (Occip_R), anterior corpus callosum (ACC), posterior corpus callosum (PCC), caudate left (Caud_L) and thalamus left (Thal_L).

Regions of interest from the Harvard Oxford subcortical and MNI structural probabilistic atlases available within FSL were applied in normalised space^43^. The ROI template was modified by erosion of a single voxel using the fslmaths tool within FSL to improve spatial localisation and reduce the impact of coregistration, normalisation and partial volume errors. In patients, these analyses were performed on “lesion free” brain by exclusion of lesion core and contusion tissue following transformation of the lesion ROI to normalised space (Fig. 4). While this resulted in the removal of a few regions were a lesion covered the entire ROI, normal appearing tissue from within the remaining volume of brain of each region was retained for subsequent analyses. The FA, MD, AD and RD values for the different ROIs were calculated using in-house software using Matlab (Mathworks, Natick, USA).

### Impact of hyperoxia

Using published DTI reproducibility data from the historical cohort of 26 volunteers included in this manuscript^34^, we assessed the significance of changes in DTI parameters following NH. Based on the standard deviation (SD) of DTI measurements the overall population 99% PIs for zero change (based on three SD values) were 9.6 × 10^−5^, 9.6 × 10^−5^ and 2.5 × 10^−4^ mm^2^/second for AD, RD and MD respectively, and 3.6 × 10^−2^ for FA^34^. We calculated the percentage of ROIs in patients with increases or decreases in DTI parameters greater than the overall population 99% PI for zero change. Since measurements of reproducibility can vary depending on the brain region examined^34^ we also used an estimate of the regional 99% prediction interval for zero change calculated for each ROI. As this is based on the four independent measurements obtained for each ROI in isolation, we must be more cautious. For a *t* distribution with 3 degrees of freedom this should be based on 5.8 SD values, and this estimate was used for each individual ROI value in calculating the regional 99% prediction interval for zero change.

### Data and statistical analysis

Statistical analyses were conducted using Statview (Version 5, 1998, SAS Institute Inc., Cary, North Carolina, USA). All data are expressed and displayed as mean and SD, unless otherwise stated. Individual ROIs were treated independently, since they represented a clinically relevant method of segmenting the brain, with specific location being irrelevant to this analysis. Data were compared using unpaired, paired t-tests, ANOVA and Mann Whitney U tests. All p values are quoted after Bonferroni correction (where appropriate), and p values that remained <0.05 following multiplication by the number of tests performed were considered significant. The datasets generated during and/or analysed during the current study are available from the corresponding author on reasonable request.

## Electronic supplementary material

Supplementary Tables
