# Supplementary material for: Normobaric hyperoxia does not improve derangements in diffusion tensor imaging found distant from visible contusions following acute traumatic brain injury
Source: Sci Rep. 2017 Sep 29;7:12419. doi: 10.1038/s41598-017-12590-2 (PMC5622132; doi:10.1038/s41598-017-12590-2)
Supplement: Supplementary file 1 — Supplementary Tables [file 41598_2017_12590_MOESM1_ESM.doc]

# Normobaric hyperoxia does not improve derangements in diffusion tensor imaging found distant from visible contusions following acute traumatic brain injury

### Tonny V Veenith,^1,2^ Eleanor L Carter,^1^ Julia Grossac,^1,3^ Virginia FJ Newcombe,^1^ Joanne G Outtrim,^1^ Sri Nallapareddy,^1^ Victoria Lupson,^4^ Marta M Correia,^4^ Marius M Mada,^4^ Guy B Williams,^4^ David K Menon,^1^ *Jonathan P Coles.^1^

|  | ***FA*** | | ***AD*** | | ***RD*** | | ***MD*** | |
| --- | --- | --- | --- | --- | --- | --- | --- | --- |
| **Subject** | **Increases** | **Decreases** | **Increases** | **Decreases** | **Increases** | **Decreases** | **Increases** | **Decreases** |
| 1 |  | VM, P right, ACC, CP left, DM, P left, CP right, C right, CT right, ATR left |  | VM, ACC, P right, CP left, P left |  |  |  |  |
| 2 |  | VM, P right, P left, ATR right, CP left, F Mi |  |  |  |  |  |  |
| 3 |  |  |  |  |  |  |  |  |
| 4 |  | VM |  | VM |  |  |  | VM |
| 5 |  | VM, CP left, ATR right, ACC, CT right, DM |  | DM, PCC, SLF R, F Ma, VM |  |  |  | PCC, UF right, DM, SLF right |
| 6 | VM, CT right, PCC, CT left, CP left, BCC, DM, SLF right, SLF left, P left | C right | PCC, CT right, CT left, VM, CP left | C right, C left |  |  | CT left, CT right, PCC | C right, C left, UF left, ATR left, UF right |
| 7 |  |  |  |  |  |  |  |  |
| 8 |  |  | VM |  |  |  |  |  |
| 9 |  |  |  |  |  |  |  |  |
| 10 |  | VM, C right, P left, CP right, CP left |  | VM, C right |  |  |  |  |
| 11 |  | VM, ATR right, ACC, P right, P left, ATR left, CP right, CP left, CT right | P right |  |  |  | P right | VM |
| 12 |  |  |  |  |  |  |  |  |
| 13 | BCC | VM, P right, ACC, C right, P right, C left, CP right, CP left, CT left, ATR left |  | VM, ACC, PCC, P left |  |  |  | VM |
| 14 |  | VM |  |  |  |  |  |  |
| **N (%) regions** | **11 (3)** | **50 (16)** | **7 (2)** | **22 (7)** | **0** | **0** | **4 (1)** | **14 (4)** |

**Supplementary Table 1. Patient white matter regions demonstrating a change following hyperoxia using the population 99% prediction interval**

Regions showing a significant increase or decrease following hyperoxia that was greater than the overall population 99% prediction interval (PI) are shown for 14 patients with head injury for fractional anisotropy (FA), mean diffusivity (MD), axial (AD) and radial diffusivity (RD). The total number of regions in this patient cohort was 320, and the number (N) and percentage (%) of this total showing a change is provided in the table. Anterior corpus callosum (ACC), body corpus callosum (BCC), posterior corpus callosum (PCC), anterior thalamic radiation (ATR), superior longitudinal fasciculus (SLF), inferior longitudinal fasciculus (ILF), Cingulum (C), uncinate fasciculus (UF), corticospinal tract (CT), forceps minor (F Mi), forceps major (F Ma), ventral midbrain (VM), dorsal midbrain (DM), cerebral peduncle (CP), pons (P).

|  | ***FA*** | | ***AD*** | | ***RD*** | | ***MD*** | |
| --- | --- | --- | --- | --- | --- | --- | --- | --- |
| **Subject** | **Increases** | **Decreases** | **Increases** | **Decreases** | **Increases** | **Decreases** | **Increases** | **Decreases** |
| 1 |  | H left, H right, Thal left, Temp right, P right, O right, O left, Thal right, Temp left |  |  |  |  |  |  |
| 2 |  | Temp right, H right, Temp left, O right |  | Caud right |  |  |  | Caud right |
| 3 |  |  |  |  |  |  |  |  |
| 4 |  |  |  |  |  |  |  | H right |
| 5 |  | Thal right, Cereb left, Thal left, Caud right, O right | Caud right | O right, P right, Temp left | Caud right |  | Caud right, Thal right | Temp right, F right |
| 6 | F left, P left, F right, Thal left, P right, Thal right | H right, Caud left | P right, P left, F right, F left, H right | Thal right, Thal left |  | Thal right, Thal left | P right, P left, H right | Thal right, Thal left, Caud right, Caud left |
| 7 |  |  |  |  |  |  |  |  |
| 8 |  | Caud left |  |  |  |  |  |  |
| 9 |  |  |  |  |  |  |  |  |
| 10 |  |  | Caud right |  |  |  |  |  |
| 11 |  | Caud left, Caud right, Thal right, Thal left |  | Caud left |  |  |  |  |
| 12 |  |  |  |  |  |  |  |  |
| 13 |  | Temp left, H left, Caud right, Caud left, F right |  |  |  |  |  |  |
| 14 |  | Caud right |  |  |  |  |  |  |
| **N (%) regions** | **6 (3)** | **31 (14)** | **7(3)** | **7 (3)** | **1 (0)** | **2 (1)** | **5(2)** | **8 (4)** |

**Supplementary Table 2. Patient mixed cortical and deep grey matter regions demonstrating a change following hyperoxia using the population 99% prediction interval**

Regions showing a significant increase or decrease following hyperoxia that was greater than the overall population 99% prediction interval (PI) are shown for 14 patients with head injury for fractional anisotropy (FA), mean diffusivity (MD), axial (AD) and radial diffusivity (RD). The total number of regions in this patient cohort was 223, and the number (N) and percentage (%) of this total showing a change is provided in the table. Caudate (Caud), thalamus (Thal), hippocampus (H), frontal (F), parietal (P), temporal (Temp), occipital (O), cerebellum (Cereb).

|  | ***FA*** | | ***AD*** | | ***RD*** | | ***MD*** | |
| --- | --- | --- | --- | --- | --- | --- | --- | --- |
| **Volunteer** | **Increases** | **Decreases** | **Increases** | **Decreases** | **Increases** | **Decreases** | **Increases** | **Decreases** |
| 1 | ACC | P left | ACC | P left |  |  |  |  |
| 2 | PCC | BCC |  | PCC |  |  |  | PCC |
| 3 | ACC |  |  |  |  |  |  |  |
| 4 |  |  |  |  |  |  |  |  |
| 5 |  | VM |  | VM |  |  |  |  |
| 6 |  | ACC, VM, P right | VM |  |  |  | VM |  |
| **N (%) regions** | **3 (2)** | **6 (4)** | **2(1)** | **3 (2)** | **0** | **0** | **1(1)** | **1 (1)** |

**Supplemental Table 3. Healthy volunteer white matter regions demonstrating a change following hyperoxia using the population 99% prediction interval**

Regions showing a significant increase or decrease following hyperoxia that was greater than the overall population 99% prediction interval (PI) are shown for 6 controls for fractional anisotropy (FA), mean diffusivity (MD), axial (AD) and radial diffusivity (RD). The total number of regions in this patient cohort was 138, and the number (N) and percentage (%) of this total showing a change is provided in the table. Anterior corpus callosum (ACC), body corpus callosum (BCC), posterior corpus callosum (PCC), anterior thalamic radiation (ATR), superior longitudinal fasciculus (SLF), inferior longitudinal fasciculus (ILF), Cingulum (C), uncinate fasciculus (UF), corticospinal tract (CT), forceps minor (F Mi), forceps major (F Ma), ventral midbrain (VM), dorsal midbrain (DM), cerebral peduncle (CP), pons (P).

|  | ***FA*** | | ***AD*** | | ***RD*** | | ***MD*** | |
| --- | --- | --- | --- | --- | --- | --- | --- | --- |
| **Volunteer** | **Increases** | **Decreases** | **Increases** | **Decreases** | **Increases** | **Decreases** | **Increases** | **Decreases** |
| 1 |  |  |  |  |  |  |  |  |
| 2 |  |  |  |  |  |  |  |  |
| 3 |  |  |  |  |  |  |  |  |
| 4 |  |  |  |  |  |  |  |  |
| 5 |  |  |  |  |  |  |  |  |
| 6 | Caud right | Caud left | Caud right |  |  |  |  |  |
| **N (%) regions** | **1 (1)** | **1 (1)** | **1(1)** | **0** | **0** | **0** | **0** | **0** |

**Supplemental Table 4. Healthy volunteer mixed cortical and deep grey matter regions demonstrating a change following hyperoxia using the population 99% prediction interval**

Regions showing a significant increase or decrease following hyperoxia that was greater than the overall population 99% prediction interval (PI) are shown for 6 controls for fractional anisotropy (FA), mean diffusivity (MD), axial (AD) and radial diffusivity (RD). The total number of regions in this patient cohort was 96, and the number (N) and percentage (%) of this total showing a change is provided in the table. Caudate (Caud), thalamus (Thal), hippocampus (H), frontal (F), parietal (P), temporal (Temp), occipital (O), cerebellum (Cereb).

|  | ***FA*** | | ***AD*** | | ***RD*** | | ***MD*** | |
| --- | --- | --- | --- | --- | --- | --- | --- | --- |
| **Subject** | **Increases** | **Decreases** | **Increases** | **Decreases** | **Increases** | **Decreases** | **Increases** | **Decreases** |
| 1 |  | ACC, ATR left, ATR right, ILF right, C right, CT right, VM, CP left, CP right, P left, P right |  | ACC, VM, CP left, P right |  |  |  | ACC |
| 2 |  | ATR right |  |  |  |  |  |  |
| 3 |  |  |  |  |  |  |  |  |
| 4 |  | VM |  |  |  |  |  |  |
| 5 |  | ATR left, ATR right, VM, CP left |  | PCC, SLF left, SLF right, ILF left, UF left, F Ma, DM | ATR right | SLF right |  | PCC, ATR left, SLF right, IFL left, IFL right, UF right, F Mi, F Ma |
| 6 | PCC, ATR left, SLF left, SLF right, CT left, CT right, VM, CP left |  | PCC, CT left, CT right, CP left | C left, C right | CT left, CT right | ATR left, C left, C right | CT left, CT right | ATR left, ATR right, ILF left, ILF right, C left, C right, UF left, UF right |
| 7 |  |  |  |  |  |  |  | ILF L, UF L, UF R |
| 8 |  |  |  |  |  |  |  |  |
| 9 |  |  |  |  |  |  |  |  |
| 10 |  | C right |  |  |  |  |  |  |
| 11 |  | ACC, ATR left, ATR right, VM, P left, P right |  | ACC, ATR left, VM |  |  |  |  |
| 12 |  |  |  |  |  |  |  |  |
| 13 |  | ATR left, C left, C right, VM, CP right, P left |  | C left |  |  |  | C left |
| 14 |  |  |  |  |  |  |  |  |
| **N (%) regions** | **8 (3)** | **30 (9)** | **4(1)** | **17 (5)** | **3 (1)** | **4 (1)** | **2(1)** | **21 (7)** |

**Supplementary Table 5. Patient white matter regions demonstrating a change following hyperoxia using the regional 99% prediction interval**

Regions showing a significant increase or decrease following hyperoxia that was greater than the regional 99% prediction interval (PI) are shown for 14 patients with head injury for fractional anisotropy (FA), mean diffusivity (MD), axial (AD) and radial diffusivity (RD). The total number of regions in this patient cohort was 320, and the number (N) and percentage (%) of this total showing a change is provided in the table. Anterior corpus callosum (ACC), body corpus callosum (BCC), posterior corpus callosum (PCC), anterior thalamic radiation (ATR), superior longitudinal fasciculus (SLF), inferior longitudinal fasciculus (ILF), Cingulum (C), uncinate fasciculus (UF), corticospinal tract (CT), forceps minor (F Mi), forceps major (F Ma), ventral midbrain (VM), dorsal midbrain (DM), cerebral peduncle (CP), pons (P).

|  | ***FA*** | | ***AD*** | | ***RD*** | | ***MD*** | |
| --- | --- | --- | --- | --- | --- | --- | --- | --- |
| **Subject** | **Increases** | **Decreases** | **Increases** | **Decreases** | **Increases** | **Decreases** | **Increases** | **Decreases** |
| 1 |  | Thal left, Thal right, H left, H right, P left, P right, Temp left, Temp right, O left, O right |  | Thal R, H left |  |  |  | Thal right |
| 2 |  | H left, H right, F left, F right, P left, Temp left, Temp right, O left, O right |  | Caud right |  | Caud right |  | Caud right |
| 3 |  |  |  |  |  |  |  |  |
| 4 |  |  |  |  |  |  |  | H right |
| 5 |  | Thal left, Thal right, F left, F right, P left, P right, O right | Caud R, Thal R | P right, Temp L, O right | Caud right, Thal right |  | Caud right, Thal right, P right, Temp right, O right |  |
| 6 | Thal left, Thal right, F left, F right, P left, P right | Caud left, H left, H right | H right, F left, F right, P left, P right, Temp R, O left, O right | Thal L, Thal R | H right, F right, P left, P right | Thal left, Thal right | H right, P left, P right | Caud right, Thal left, Thal right |
| 7 |  |  |  |  |  |  |  |  |
| 8 |  |  |  |  |  |  |  |  |
| 9 |  |  |  |  |  |  |  |  |
| 10 |  | P left, O right |  |  |  |  |  |  |
| 11 |  | Caud left, Caud right, Thal left, Thal right |  |  |  |  |  |  |
| 12 |  |  |  |  |  |  |  |  |
| 13 |  | H left, F left, F right, P left, Temp left, O left, O right |  |  |  |  |  |  |
| 14 |  | O right |  |  |  |  |  |  |
| **N (%) regions** | **6 (3)** | **43 (19)** | **10(4)** | **8 (4)** | **6 (3)** | **3 (1)** | **8(4)** | **6 (3)** |

**Supplementary Table 6. Patient mixed cortical and deep grey matter regions demonstrating a change following hyperoxia using the regional 99% prediction interval**

Regions showing a significant increase or decrease following hyperoxia that was greater than the regional 99% prediction interval (PI) are shown for 14 patients with head injury for fractional anisotropy (FA), mean diffusivity (MD), axial (AD) and radial diffusivity (RD). The total number of regions in this patient cohort was 223, and the number (N) and percentage (%) of this total showing a change is provided in the table. Caudate (Caud), thalamus (Thal), hippocampus (H), frontal (F), parietal (P), temporal (Temp), occipital (O), cerebellum (Cereb).

|  | ***FA*** | | ***AD*** | | ***RD*** | | ***MD*** | |
| --- | --- | --- | --- | --- | --- | --- | --- | --- |
| **Volunteer** | **Increases** | **Decreases** | **Increases** | **Decreases** | **Increases** | **Decreases** | **Increases** | **Decreases** |
| 1 |  |  | ACC | ILF left, P left |  |  | ACC | ILF left |
| 2 | PCC |  |  | PCC |  | PCC | P left | PCC, CP right |
| 3 |  |  |  |  |  |  |  |  |
| 4 |  |  |  |  |  |  |  |  |
| 5 |  |  |  |  |  |  |  |  |
| 6 |  |  |  |  | VM |  | P right |  |
| **N (%) regions** | **1 (1)** | **0** | **1 (1)** | **3 (2)** | **1 (1)** | **1 (1)** | **3(2)** | **3 (2)** |

**Supplementary Table 7. Healthy volunteer white matter regions demonstrating a change following hyperoxia using the regional 99% prediction interval**

Regions showing a significant increase or decrease following hyperoxia that was greater than the regional 99% prediction interval (PI) are shown for 6 controls for fractional anisotropy (FA), mean diffusivity (MD), axial (AD) and radial diffusivity (RD). The total number of regions in this patient cohort was 138, and the number (N) and percentage (%) of this total showing a change is provided in the table. Anterior corpus callosum (ACC), body corpus callosum (BCC), posterior corpus callosum (PCC), anterior thalamic radiation (ATR), superior longitudinal fasciculus (SLF), inferior longitudinal fasciculus (ILF), Cingulum (C), uncinate fasciculus (UF), corticospinal tract (CT), forceps minor (F Mi), forceps major (F Ma), ventral midbrain (VM), dorsal midbrain (DM), cerebral peduncle (CP), pons (P).

|  | ***FA*** | | ***AD*** | | ***RD*** | | ***MD*** | |
| --- | --- | --- | --- | --- | --- | --- | --- | --- |
| **Volunteer** | **Increases** | **Decreases** | **Increases** | **Decreases** | **Increases** | **Decreases** | **Increases** | **Decreases** |
| 1 |  |  | Temp right |  |  |  | Temp right |  |
| 2 |  |  |  |  |  |  |  |  |
| 3 |  |  |  |  |  |  |  |  |
| 4 |  |  | Thal right |  |  |  | Thal right |  |
| 5 |  |  |  |  |  |  |  |  |
| 6 |  | Caud left | Caud right, P right |  |  |  | P right |  |
| **N (%) regions** | **0** | **1 (1)** | **4 (4)** | **0** | **0** | **0** | **3(3)** | **0** |

**Supplementary Table 8. Healthy volunteer mixed cortical and deep grey matter regions demonstrating a change following hyperoxia using the regional 99% prediction interval**

Regions showing a significant increase or decrease following hyperoxia that was greater than the regional 99% prediction interval (PI) are shown for 6 controls for fractional anisotropy (FA), mean diffusivity (MD), axial (AD) and radial diffusivity (RD). The total number of regions in this patient cohort was 96, and the number (N) and percentage (%) of this total showing a change is provided in the table. Caudate (Caud), thalamus (Thal), hippocampus (H), frontal (F), parietal (P), temporal (Temp), occipital (O), cerebellum (Cereb).
